# Local adaptation in European populations affected the genetics of psychiatric disorders and behavioral traits

**DOI:** 10.1186/s13073-018-0532-7

**Published:** 2018-03-26

**Authors:** Renato Polimanti, Manfred H. Kayser, Joel Gelernter

**Affiliations:** 1 Department of Psychiatry, Yale School of Medicine and VA CT Healthcare Center, 950 Campbell Avenue, West Haven, CT 06516 USA; 2 Department of Genetic Identification, Erasmus University Medical Center, Rotterdam, Rotterdam, the Netherlands; 3 Departments of Genetics and Neuroscience, Yale School of Medicine, New Haven, CT USA

**Keywords:** Evolution, Natural selection, Genetic diversity, GWAS, Polygenicity, Psychiatry

## Abstract

**Background:**

Recent studies have used genome-wide data to investigate evolutionary mechanisms related to behavioral phenotypes, identifying widespread signals of positive selection. Here, we conducted a genome-wide investigation to study whether the molecular mechanisms involved in these traits were affected by local adaptation.

**Methods:**

We performed a polygenic risk score analysis in a sample of 2455 individuals from 23 European populations with respect to variables related to geo-climate diversity, pathogen diversity, and language phonological complexity. The analysis was adjusted for the genetic diversity of European populations to ensure that the differences detected would reflect differences in environmental exposures.

**Results:**

The top finding was related to the association between winter minimum temperature and schizophrenia. Additional significant geo-climate results were also observed with respect to bipolar disorder (sunny daylight), depressive symptoms (precipitation rate), major depressive disorder (precipitation rate), and subjective well-being (relative humidity). Beyond geo-climate variables, we also observed findings related to pathogen diversity and language phonological complexity: openness to experience was associated with protozoan diversity; conscientiousness and extraversion were associated with language consonants.

**Conclusions:**

We report that common variation associated with psychiatric disorders and behavioral traits was affected by processes related to local adaptation in European populations.

**Electronic supplementary material:**

The online version of this article (10.1186/s13073-018-0532-7) contains supplementary material, which is available to authorized users.

## Background

Recent studies have used genome-wide data to investigate evolutionary mechanisms related to behavioral phenotypes, identifying widespread signals of positive selection (i.e., variants with beneficial effects on individual fitness increase in population frequency) in the predisposition to psychiatric disorder and behavioral traits [1–3]. Brain-related phenotypes have undergone polygenic adaptation (adaptation that occurs by simultaneous selection on variants at many loci) during different phases of human evolutionary history [4] including to the present day [5]. This is consistent with several other investigations that found evidence of polygenic adaptation for predisposition to a wide range of complex traits [6–9]. These genome-wide signals of positive selection are the signatures of adaptation processes that occurred in response to environmental pressures. Single-variant analyses identified loci affected by local adaption (i.e., adaptation in response to selective pressure related to the local environment) to diet, pathogens, and geo-climate variables [10, 11]. Polygenic mechanisms have also been observed in response to local environments. The observed difference in height between northern and southern Europeans appears to be related to a highly polygenic mechanism [12]. Polygenic risk scores (PRSs) for height, skin pigmentation, body mass index, type 2 diabetes, Crohn’s disease, and ulcerative colitis were tested with respect to geo-climate variables in worldwide populations, with the discovery of putative signals of local adaptation [9]. However, a recent analysis demonstrated that PRSs derived from genome-wide association studies (GWASs) on populations of European descent generate biased results when applied to non-European samples [13]. PRS analysis should thus be limited to training and target datasets with the same ancestry backgrounds; we were therefore able to investigate local adaption only in European populations. To investigate whether molecular mechanisms at the basis of psychiatric/behavioral traits (Table 1) were affected by local-adaptation processes that occurred during the colonization of Europe [14], we conducted a PRS analysis based on GWASs of psychiatric disorders and behavioral traits (Table 1) from the Psychiatric Genomics Consortium [15–17], the Genetics of Personality Consortium [18–20], and the Social Science Genetic Association Consortium [21] in a sample of 2455 individuals from 23 European populations. Then, we conducted a Gene Ontology (GO) enrichment analysis based on PRS results to provide information regarding the specific molecular mechanisms involved in the polygenic signatures of local adaptation observed.

**Table 1:** GWASs of psychiatric disorders and behavioral traits used to generate polygenic risk scores

Consortium	Trait	Abbreviation	Sample size	Publication year	Link
Psychiatric Genomics Consortium	Autism spectrum disorder	ASD	5305 cases	2015	https://www.med.unc.edu/pgc/results-and-downloads
	Bipolar disorder	BD	7481 cases	2011	
	Major depressive disorder	MDD	9240 cases	2013	
	Schizophrenia	SCZ	36,989 cases	2014	
Genetics of Personality Consortium	Neuroticism	gpcNEURO	63,661	2015	http://www.tweelingenregister.org/GPC/
	Extraversion	EXTRA	63,030	2016	
	Openness to experience	OPEN	17,375	2012	
	Agreeableness	AGREE	17,375	2012	
	Conscientiousness	CONS	17,375	2012	
Social Science Genetic Association Consortium	Subjective well-being	SWB	298,420	2016	https://www.thessgac.org/data
	Depressive symptoms	DS	161,460	2016	
	Neuroticism	ssgacNEURO	170,911	2016	

## Methods

### Study population

The cohort used in the present study was previously investigated to analyze the genetic structure of European populations [22]. The sample included individuals from 23 different sampling sites located in one of 20 different European countries (Additional file 1: Table S1). The GeneChip Human Mapping 500K Array Set (Affymetrix) was used to genotype 500,568 single nucleotide polymorphisms (SNPs) according to the instructions provided by the manufacturer as reported previously [22]. The analysis of identity-by-state values permitted us to exclude the possibility of the presence of related individuals (i.e., individuals who were genetically more similar than expected to another member of the same subpopulation) and outliers (i.e., individuals who were far less genetically similar than expected to the rest of the subpopulation). We used this genotype information for imputation to maximize a consistent SNP panel between this cohort and the GWAS summary statistics used for the PRS analysis. Pre-imputation quality control criteria were minor allele frequency ≥ 1%, missingness per marker ≤ 5%, missingness per individual ≤ 5%, and Hardy-Weinberg equilibrium *p* > 10^−4^. We used SHAPEIT [23] for pre-phasing, IMPUTE2 [24] for imputation, and the 1000 Genomes Project reference panel [25]. We retained imputed SNPs with high imputation quality (genotype call probability ≥ 0.8), minor allele frequency ≥ 1%, missingness per marker ≤ 5%, and missingness per individual ≤ 5%. After applying the post-imputation quality control criteria, we retained information regarding 3,416,230 variants in a final sample of 2455 individuals. Principal component analysis of the final sample was conducted using PLINK 1.9 [26] after linkage disequilibrium (LD) pruning (*R*^2^ < 0.2) of the genotyped data. Principal components derived from genetic information were included in the regression model to adjust the analysis for population genetic background, which reflects the demographic history of European populations [27]. In line with previous PRS analyses [28–32], the initial analysis was conducted including the top 10 principal components. To verify whether residual population stratification affected our analysis, the top 20 principal components were included as covariates to confirm the reliability of the significant findings.

### Local-adaptation variables

We extracted information regarding local adaptation by considering the location of the 23 sampling sites used to recruit the cohort investigated. Specifically, we considered three different types of variables: geo-climate (geographical coordinates, temperature, daylight, precipitation rate, and humidity), pathogen diversity (bacteria, protozoa, and virus), and language phonological complexity (consonants, segments, and vowels) (Table 2). Geo-climate information was extracted from ClimaTemps (available at http://www.climatemps.com/), which contains more than 12.5 million climate comparison reports providing information for more than 4000 locations worldwide. Data regarding pathogen diversity were extracted from the GIDEON (Global Infectious Diseases and Epidemiology Online Network) database (available at https://www.gideononline.com/). This includes information regarding 350 infectious diseases and 1700 microbial taxa in 231 countries. Information about the phonological complexity of European languages was extracted from PHOIBLE Online (available at http://phoible.org/), which is a repository of cross-linguistic phonological inventory data including 2155 inventories that contain 2160 segment types found in 1672 distinct languages [33]. Correlations among local-adaptation variables were estimated using Spearman’s correlation test.

**Table 2:** Variables related to local adaptation tested

Category	Variable	Abbreviation	Source	Link
Geo-climate	Latitude	LAT	ClimaTemps	http://www.climatemps.com/
	Longitude	LON		
	Altitude	ALT		
	Summer temperature (Max-Min)	SumMaxTemp SumMinTemp		
	Winter temperature (Max-Min)	WinMaxTemp WinMinTemp		
	Precipitation rate (Max-Min)	MaxPrecipRate MinPrecipRate		
	Relative humidity (Max-Min)	MaxRelHumidity MinRelHumidity		
	Sunny daylight (Max-Min)	MaxSunnyDaylight MinSunnyDaylight		
Pathogen diversity	Virus	VirusDiversity	GIDEON	https://www.gideononline.com/
	Bacteria	BacteriaDiversity		
	Protozoa	ProtozoaDiversity		
Language phonological complexity	Segments	Segments	PHOIBLE	http://phoible.org/
	Vowels	Vowels		
	Consonants	Consonants		

### Polygenic risk score analysis

We conducted a PRS analysis using PRSice software [34] (available at http://prsice.info/). For polygenic profile scoring, we used summary statistics generated from multiple large-scale GWASs of psychiatric disorders and behavioral traits (Table 1) conducted by the Psychiatric Genomics Consortium [15–17], the Genetics of Personality Consortium [18–20], and the Social Science Genetic Association Consortium [21]. None of the GWASs used in the present study showed evidence of inflation due to population stratification or other possible confounders. Since none of the samples included in our target dataset was used in the GWAS considered to generate the PRS, no systematic overlap is expected between training and target datasets. We considered multiple association *p*-value thresholds (*PT* = 5 × 10^−8^, 10^−7^, 10^−6^, 10^−5^, 10^−4^, 0.001, 0.01, 0.05, 0.1, 0.3, 0.5, 1) for SNP inclusion and calculated multiple PRSs for each trait investigated. The PRSs were calculated after using *p*-value-informed clumping with an LD cutoff of *R*^2^ = 0.3 within a 500-kb window, and excluding the major histocompatibility complex region of the genome because of its complex LD structure. The PRSs that were generated were fitted in regression models with adjustments for the top 10 ancestry principal components. Before being entered into the analysis, local-adaptation variables were normalized using appropriate Box-Cox power transformations to avoid biases due to the distribution of the phenotypes tested. We applied a false discovery rate (FDR) correction (*q* < 0.05) to correct for the multiple testing for the psychiatric/behavioral PRS × local-adaptation variables tested [35]. To verify that no systematic bias inflated our analyses, we also conducted a permutation analysis. Specifically, considering the significant datasets, we performed 10,000 permutations of the PRSs with respect to their associated variables and verified whether the observed differences were significantly different from the null distribution of the permuted results. To estimate the genetic correlation among psychiatric disorders and behavioral traits, we considered the information provided by LD Hub v1.3.1 [36] (available at http://ldsc.broadinstitute.org/ldhub/) and used the LD score regression method [37] for the missing pair-wise comparisons. Heritability statistics of the GWAS considered are reported in Additional file 2: Table S2.

### Gene Ontology enrichment analysis

To provide information regarding the molecular mechanisms involved in the signatures of local adaptation in psychiatric and behavioral traits, a GO enrichment analysis was conducted based on the PRS results; the variants included in the significant PRS and with nominally significant concordant direction with PRS direction were considered in the enrichment analysis. A description of the GO analysis based on PRS results was reported in previous studies [28–30]. Variants were then entered in the enrichment analysis performed using eSNPO [38]. This method permits one to conduct enrichment analysis based on information related to expression quantitative trait loci (eQTLs) rather than physical positions of SNPs and genes, integrating the eQTL data and GO, constructing associations between SNPs and GO terms, and then performing functional enrichment analysis. An FDR correction was applied to the enrichment results for multiple testing (*q* < 0.05). To validate the results further, we conducted a permutation analysis based on the variants obtained from the major depressive disorder (MDD)-altitude result (the one that gave the highest number of significant GO enrichments). Based on this SNP set, we generated 100 SNP sets using SNPsnap (available at https://data.broadinstitute.org/mpg/snpsnap/match_snps.html) [39] and the following matching criteria: minor allele frequency ± 5%, gene density ± 50%, distance to nearest gene ± 50%, LD independence (*R*^2^ = 0.3) ± 50%. The SNP sets generated were entered in the eSNPO analysis and the distribution of their results compared with those obtained from the SNP sets from the PRS analyses.

### Natural and Orthogonal InterAction (NOIA) model

The NOIA model [40] was applied to validate the results related to single-locus and oligogenic signals identified by our PRS analysis. NOIA is able to estimate the interaction between genes (or epistasis), which is a key process in determining the effect of genomic variants in complex diseases and the adaptation and evolution of natural populations [41]. We performed NOIA analysis testing the genotypes of the variants included in the significant PRSs with respect to local-adaptation variables identified. The NOIA analysis was conducted using the R package *noia* (available at https://cran.r-project.org/web/packages/noia/index.html).

### Data sources

Data supporting the findings of this study are available within this article and its additional files. GWAS summary association data used to calculate PRSs in this study were obtained from the Psychiatric Genomics Consortium (available at https://www.med.unc.edu/pgc/results-and-downloads/), the Genetics of Personality Consortium (available at http://www.tweelingenregister.org/GPC/), and the Social Science Genetic Association Consortium (available at https://www.thessgac.org/data).

## Results

**Fig. 1:**
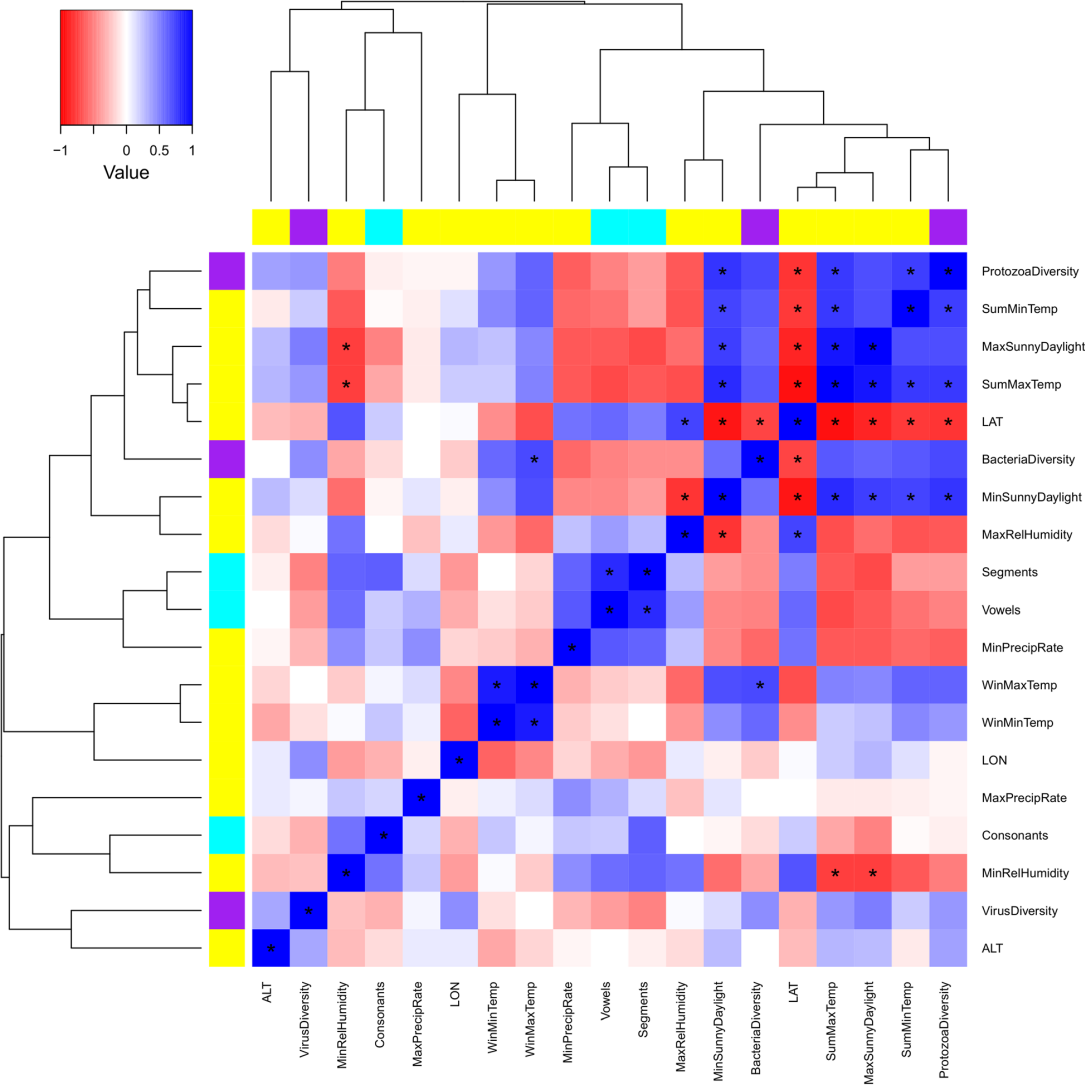
Correlations (Spearman’s rho) among variables related to local adaptation (*left*). Abbreviations are reported in Table 1 and Table 2. Additional file 3: Table S3 reports details of the correlation analysis. *Asterisks* (*) indicate correlations surviving Bonferroni multiple testing correction. *Yellow*, *purple*, and *cyan* colors indicate variables related to geo-climate, pathogen diversity, and language phonological complexity, respectively. Hierarchical clustering based on Spearman’s rho was generated considering absolute correlation distances

**Fig. 2:**
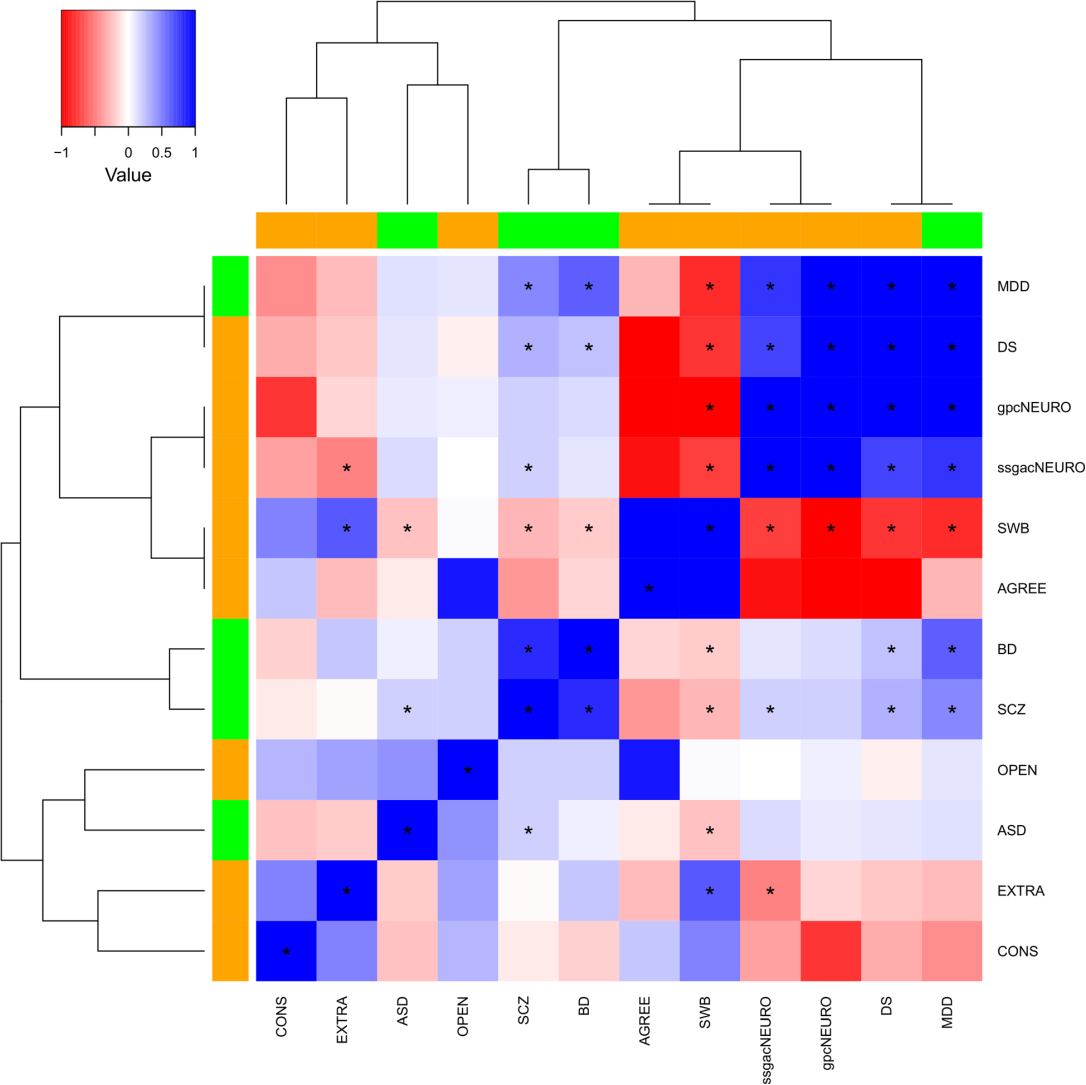
Genetic correlation (linkage disequilibrium score regression *r*_g_) among psychiatric disorders and behavioral traits (*right*). Additional file 4: Table S4 reports details of the correlation analysis. Abbreviations are reported in Table 1 and Table 2. *Asterisks* (*) indicate correlations surviving Bonferroni multiple testing correction. *Green* and *orange* colors indicate psychiatric disorders and behavioral traits, respectively. Hierarchical clustering based on genetic correlation was generated considering absolute correlation distances

As expected, the set of variables related to the local environment were strongly intercorrelated (Fig. 1; Additional file 3: Table S3). Similarly, psychiatric disorders and behavioral traits showed strong genetic correlations (Fig. 2; Additional file 4: Table S4). We considered multiple GWAS significance thresholds to test PRSs [34], investigating both oligogenic and polygenic mechanisms (i.e., local-adaptation processes affecting few and many loci, respectively). To adjust our analysis for population genetic background, which reflects the demographic history of European populations [27], we included the top 10 principal components reflecting population ancestry variation as covariates in the regression models. This approach was considered on the basis of the experience of many GWAS and PRS analyses conducted on samples containing populations of different European descents. The use of 10 principal components is generally considered a standard approach to adjust within ancestry population stratification. However, to demonstrate that our findings are not due to the genetic relationships among European populations, we recalculated the significant PRS results (Table 3) considering 20 principal components in the regression models, and then tested for differences with respect to the original model: we did not observe significant differences between the two models (Additional file 5: Table S5).

**Table 3:** Top significant associations of psychiatric and behavioral polygenic risk scores (PRSs) with the 13 local-adaptation variables identified. Abbreviations are reported in Table 1 and Table 2

Local-adaptation variable	PRS	PT	SNP N	*R* ^2^	Z score	*p* value	*q* value
MaxSunnyDaylight	BD	0.01	2833	0.09%	−2.93	3.42 × 10^−3^	0.043
Consonants	CONS	0.5	60,620	0.28%	−2.97	2.98 × 10^−3^	0.043
Latitude	DS	10^−7^	1	0.09%	3.47	5.38 × 10^−4^	0.029
SumMaxTemp				0.12%	−3.40	6.91 × 10^−4^	0.029
MinPrecipRate		0.05	12,832	0.27%	−3.29	1.03 × 10^−3^	0.029
MaxPrecipRate	MDD	0.3	39,390	0.31%	−3.21	1.33 × 10^−3^	0.034
Altitude		1	97,481	0.31%	−3.13	1.79 × 10^−3^	0.037
ProtozoaDiversity	OPEN	10^−6^	2	0.18%	3.56	3.82 × 10^−4^	0.029
SumMinTemp		5 × 10^−8^	1	0.18%	2.7	3.02 × 10^−3^	0.043
WinMinTemp	SCZ	0.5	104,106	0.40%	3.84	1.28 × 10^−4^	0.029
Longitude				0.13%	−3.29	1.01 × 10^−3^	0.029
WinMaxTemp				0.12%	2.96	3.09 × 10^−3^	0.043
MinRelHumidity	SWB	10^−6^	4	0.21%	−2.95	3.22 × 10^−3^	0.043

**Fig. 3:**
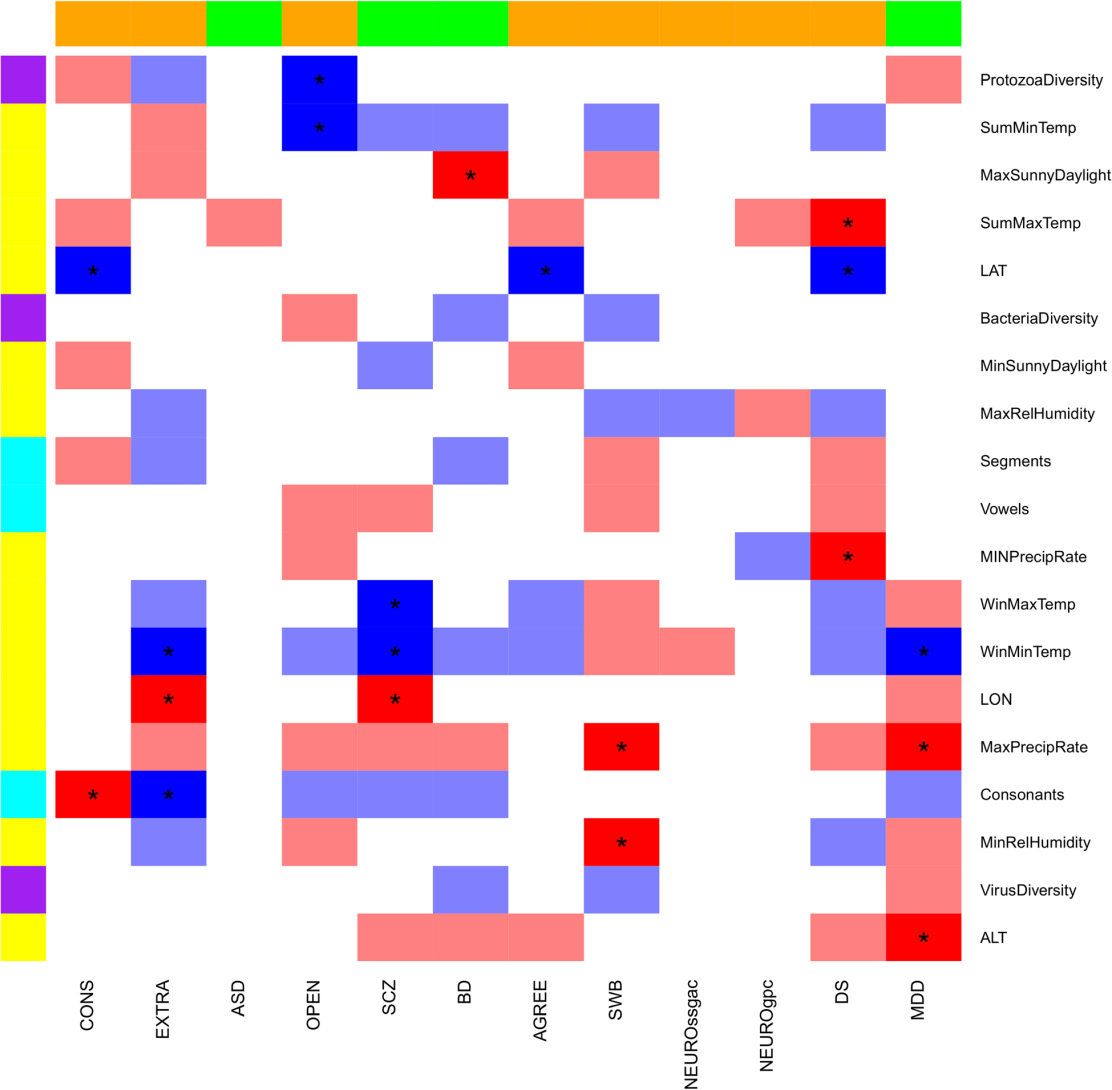
PRS results (Z scores) for psychiatric/behavioral traits × local-adaptation variables. Positive and negative associations are indicated in *blue* and *red*, respectively (*bright shade q* < 0.05, *light shade p* < 0.05). *White cells* indicate associations with *p* > 0.05. Color schemes for local-adaptation variables and psychiatric/behavioral traits are reported in the legends of Fig. 1 and Fig. 2, respectively. Abbreviations are reported in Table 1 and Table 2. Additional file 6: Table S6 reports the summary statistics of the PRS analysis

Considering the results that survived FDR multiple testing correction (*q* < 0.05; Additional file 6: Table S6), we observed 13 variables related to local adaptation: 11 geo-climate variables, one related to pathogen diversity, and one related to language phonological complexity. Table 3 reports the top associations that survived FDR multiple testing correction for each of these 13 local-adaptation variables. Figure 3 reports full visualization of the results for all comparisons (psychiatric/behavioral PRS × local-adaptation variables). We confirmed the reliability of the significant results empirically by generating a null distribution from 10,000 permutations of the original datasets and comparing the permuted results with the observed ones (Additional file 7: Figure S1). Since polygenic signatures of local adaptation have previously been reported in height genetics of European populations [12], we used this trait as a positive control for our approach. With this analysis, we replicated the presence of adaptation signals in the genetics of this trait (*p* < 0.05; Additional file 8: Table S7).

The strongest result was observed between the schizophrenia (SCZ) PRS and winter minimum temperature (WinMinTemp): higher WinMinTemp correlates with increased SCZ genetic risk (SNP *N* = 104,106, Nagelkerke’s *R*^2^ = 0.40%, *Z* = 3.84, *p* = 1.28 × 10^−4^, *q* = 0.029). Higher WinMinTemp was also associated with increased MDD PRS (SNP *N* = 8160, Nagelkerke’s *R*^2^ = 0.30%, *Z* = 3.34, *p* = 8.46 × 10^−4^, *q* = 0.029) and increased extraversion PRS (SNP *N* = 7, Nagelkerke’s *R*^2^ = 0.26%, *Z* = 3.14, *p* = 1.75 × 10^−3^, *q* = 0.037). While the MDD result is concordant with the SCZ-MDD genetic correlation, the extraversion finding seems to be independent of the SCZ and MDD results. The SCZ PRS was also associated with winter maximum temperature (WinMaxTemp) and longitude; the three environmental variables are highly correlated, and the results are driven by the same mechanism related to winter temperature. Covarying these three local-adaptation variables, WinMaxTemp appears to be the driving signal among the correlated results (*p* < 0.05; Additional file 9: Table S8).

To understand better the molecular processes involved in this association, we conducted a GO enrichment analysis based on the PRS result. We observed 16 GOs that survived FDR multiple testing correction (*q* < 0.05; Additional file 10: Table S9). Among the other significant PRS associations, we observed significant GO enrichments (*N* = 54; Additional file 11: Table S10) in the negative association between altitude and MDD PRS (SNP *N* = 97,481, Nagelkerke’s *R*^2^ = 0.31%, *Z* = −3.13, *p* = 1.79 × 10^−3^, *q* = 0.037) only. Five GO enrichments are significant in both SCZ and MDD analyses (GO:0008285~negative regulation of cell proliferation, GO:0017147~Wnt-protein binding, GO:2000041~negative regulation of planar cell polarity pathway involved in axis elongation, GO:0071481~cellular response to X-ray, and GO:0090244~Wnt signaling pathway involved in somitogenesis); two of these are related to the Wnt signaling pathway. To confirm empirically that these enrichment results are not false positives, we conducted a permutation analysis: we generated 100 random sets of LD-independent variants derived from the SNPs included in the MDD analysis (which was the one that gave the highest number of GO enrichments), considering minor allele frequency, gene density, distance to nearest gene, and LD independence as matching criteria. There was no permuted set with more than two significant GO enrichments (i.e., the empirical probability to observe a random set with more than two significant GO enrichments is *p* < 0.01; Additional file 12: Figure S2); the overall probability to observe a significant GO enrichment from a permuted set is *p* = 6.69*10^−5^ (Additional file 13: Figure S3); and none of the four GOs shared by SCZ and MDD results resulted in significance in the permuted sets (*q* > 0.18).

Among the psychiatric disorders investigated, MDD and depressive symptoms (DS) showed a very strong genetic correlation (*r*_g_ = 1, *p* = 1.77 × 10^−36^). In accordance with this genetic overlap, we observed a convergence in the local-adaptation findings that survived multiple testing correction. The MDD and DS PRS showed concordant negative associations with precipitation rate (PR): maximum PR (SNP *N* = 39,390, Nagelkerke’s *R*^2^ = 0.31%, *Z* = −3.21, *p* = 1.33 × 10^−3^, *q* = 0.034) and minimum PR (SNP *N* = 12,832, Nagelkerke’s *R*^2^ = 0.27%, *Z* = −3.29, *p* = 1.03 × 10^−3^, *q* = 0.029), respectively. The same DS PRS also nominally replicated the negative association with maximum PR (SNP *N* = 12,832, Nagelkerke’s *R*^2^ = 0.16%, *Z* = −2.28, *p* = 0.022).

An additional polygenic signature of local adaptation was observed between bipolar disorder (BD) and maximum sunny daylight, where increased daylight is associated with reduced BD genetic risk (SNP *N* = 2833, Nagelkerke’s *R*^2^ = 0.09%, *Z* = −2.93, *p* = 3.42 × 10^−3^, *q* = 0.043).

The results discussed above are related to highly polygenic local-adaptation mechanisms (i.e., thousands of variants involved). However, we also observed some instances of local adaptation involving few loci. Among them, the strongest signal was the positive association between protozoa diversity and openness-to-experience (OPEN) score including the top two associated variants (rs1477268 and rs10932966; SNP *N* = 2, Nagelkerke’s *R*^2^ = 0.18%, *Z* = 3.56, *p* = 3.82 × 10^−4^, *q* = 0.029). An OPEN score including only rs1477268 showed a positive association with summer minimum temperature (SNP *N* = 1, Nagelkerke’s *R*^2^ = 0.18%, *Z* = 2.7, *p* = 3.02 × 10^−3^, *q* = 0.043). Another single-locus result was observed between rs6992714, which is associated with DS risk, and latitude (SNP *N* = 1, Nagelkerke’s *R*^2^ = 0.09%, *Z* = 3.47, *p* = 5.38 × 10^−4^, *q* = 0.029) and summer maximum temperature (SNP *N* = 1, Nagelkerke’s *R*^2^ = 0.12%, *Z* = −3.40, *p* = 6.91 × 10^−4^, *q* = 0.029). According to GTEx data [42], rs6992714 is associated with *GGH* (*gamma-glutamyl hydrolase*) gene expression (beta = −0.13, *p* = 3.3 × 10^−5^; Additional file 14: Figure S4). NOIA analysis confirmed the presence of additive effects in the models based on the single-locus and oligogenic PRS with respect to the local-adaptation variables identified as significant (*p* < 0.05; Additional file 15: Table S11).

Finally, we observed a genetic association with respect to language phonological complexity: the number of consonants in European languages is positively associated with genome-wide PRS of conscientiousness (SNP *N* = 60,620, Nagelkerke’s *R*^2^ = 0.28%, *Z* = −2.97, *p* = 2.98 × 10^−3^, *q* = 0.043) and extraversion (SNP *N* = 3261, Nagelkerke’s *R*^2^ = 0.26%, *Z* = 2.87, *p* = 4.13 × 10^−3^, *q* = 0.049).

## Discussion

There are many datasets available with information regarding positive selection signatures in reference European populations [43, 44]. We previously used these available data, observing a significant enrichment for positive selection in the genetics of psychiatric disorders [1]. Comparable results have been observed by independent groups using different approaches [2, 3]. Our current analysis provides novel data with respect to local-adaptation differences among European populations. Indeed, considering positive selection signals in a reference European population, the signatures of positive selection are those shared by European populations and those specific for that particular population. With local-adaptation analysis, we are investigating the differences in selective pressures among a set of distinct European populations. Thus, the signals detected in the reference population may not overlap with those related to the local-adaptation mechanisms. To be able to use tests for positive selection (e.g., haplotype-based methods), we would need a larger sample in each of the populations considered.

Our PRS analysis identified 20 associations that survived FDR multiple testing correction (Additional file 5: Table S5). The specific characteristics of the sample investigated may generate false positive results due to several factors (e.g., different sample sizes at the different populations and non-random spatial sampling). However, our permutation analysis of the significant PRS results (i.e., we permuted the genetic scores with respect to the environmental variables) indicated that there is little possibility of bias due to the composition of the sample investigated.

Our findings appear to indicate that psychiatric and behavioral traits are not necessarily the outcomes selected by evolutionary pressures; some of the molecular pathways involved in their predisposition were affected by local adaptation. We observed some convergence between our local-adaptation findings and known epidemiological evidence. However, our findings should be related to evolutionary forces that acted on a population level, while epidemiological evidence should be due to mechanisms that acted on an individual level. We hypothesize that evolutionary forces shaped the genetic diversity of European populations, while individual-level changes should be due to post-genetic changes (e.g., epigenetic modifications) or the interaction of social-psychological risk factors on loci affected by local adaptation.

The strongest result observed between the SCZ PRS and WinMinTemp is in line with previous epidemiological studies. Season of birth is a widely recognized SCZ risk factor, where there is significantly increased risk associated with winter birth [45]. Our current finding may justify a molecular hypothesis: loci associated with increased SCZ risk may have undergone local adaptation related to winter conditions. The same environmental pressure may be responsible for the winter-birth risk through epigenetic mechanisms in line with the convergence between regional DNA methylation changes and signals of local adaptation reported for other loci [46]. Our GO enrichment analysis highlighted Wnt signaling as one of the molecular processes affected by this local-adaptation mechanism. This biological pathway is well studied in relation to both psychiatric disorders and human evolution; synaptic Wnt signaling is implicated as a possible contributor to several major psychiatric disorders due its involvement in neural differentiation processes [47]. Signatures of positive selection were reported in relation to the Wnt signaling pathway in multiple species [48]. Our present findings indicate that risk loci for psychiatric disorders involved in this molecular pathway could have been under local adaptation in European populations.

Another result in line with a known epidemiological association is the negative association between maximum sunny daylight period and BD (bipolar disorder) PRS. Seasonality of BD symptoms is common and, in particular, light exposure during early life may have important consequences for those who are susceptible to bipolar disorder [49]. More generally, lack of daylight is implicated in mood change in seasonal affective disorder [50]. Our finding indicates that daylight may have acted as a local selective pressure with respect to molecular pathways involved in BD pathogenesis.

As mentioned above, we also observed some instances of local adaptation involving oligogenic and single-locus signals. Although top results from GWASs of psychiatric and behavioral traits do not explain a large percentage of the variance, loci surviving stringent significance cutoffs usually show larger effect sizes, suggesting that they may be involved in key mechanisms involved in the pathogenesis of the traits investigated. Among the oligogenic signals, the strongest finding is the association of OPEN PRS, including the top two associated variants (rs1477268 and rs10932966), with protozoa diversity and summer minimum temperature. These two results appear concordant with the strong positive correlation between summer minimum temperature and protozoa diversity (Spearman’s rho = 0.75, *p* = 4.51 × 10^−5^), which is consistent with the relationship between temperature and pathogen diversity [51]. rs1477268 is located near *RAS1*, which was implicated by previous studies as being involved in pathogen response [52]. From GTEx data [42], rs10932966 is significantly associated with *RP11-16P6.1* gene expression in multiple human tissues (Additional file 16: Table S12), but no information regarding its function is available. We hypothesize that these loci have been under local selective adaption in response to pathogen-related selective pressure. This is in line with the consistent literature regarding the role of selective pressures induced by pathogen diversity in shaping human genome diversity [6].

Another single-locus result was observed between rs6992714, which is associated with DS risk, with latitude and summer maximum temperature. This genetic variant is associated with *GGH* gene expression, which was previously implicated as involved in the pathogenesis of tropical sprue, a malabsorption syndrome commonly found in tropical regions [53]. According to our data, *GGH* may have been under local adaptation in relation to selective pressures induced by summer temperatures. The associations discussed appear to be related to the effect of selective pressures induced by geo-climate and pathogen-related variables on the human genome.

The relationship between genetic and language diversities has been investigated from several perspectives [54], and genetic associations with language phonological complexity require careful consideration. Our data indicate that there is at least a partial relationship between genetic variation and language diversity that is not driven by their shared association with human demographic history (which should be reflected by the genetic diversity accounted for by the adjustment for principal components derived from genetic data). This supports two possible converse scenarios: (1) genetic variation may have contributed to shape European language diversity; (2) European language diversity may have been a local selective pressure that shaped the genetics of behavioral traits. Although it is not possible to establish causality or a mechanism based on our current data, phonological working memory appears to be associated with extraversion and conscientiousness [55], in agreement with the relationship highlighted by our results.

## Conclusions

We report the first evidence regarding the role of local adaptation in shaping the genetic architecture of psychiatric disorders and behavioral traits. We hypothesize that most of our findings are due to the effects of local selective pressure on molecular pathways involved in the predisposition to these complex traits. Due to the presence of pervasive pleiotropy among them, some of the “evolutionary selected” pathways (e.g., the Wnt signaling pathway identified in the present study) are shared by multiple traits. Although our analysis was adjusted for human demographic history through principal components, we cannot exclude that genes involved in behavioral traits may have had a role in population migrations. Further analyses will be needed to explore this hypothesis. The main limitation of our current investigation is the impossibility of investigating local-adaptation mechanisms in non-European populations due to the general lack of large GWASs in individuals of African, Middle Eastern, Central Asian, East Asian, Native American, and Oceanic descents. Additionally, larger target cohorts with more individuals per population and more populations may permit one to detect further signals of local adaptation in the genetics of psychiatric and behavioral traits.

## Additional files

Additional file 1: **Table S1.** Details of the European samples investigated. (DOCX 12 kb)

Additional file 2: **Table S2.** Heritability statistics (LD score regression) of the GWASs considered. (DOCX 12 kb)

Additional file 3: **Table S3.** Correlations (Spearman’s rho, upper triangular; *p* value, lower triangular) among variables related to local adaptation. *p* values surviving Bonferroni multiple testing correction are reported in *red*. Abbreviations are reported in Table 1 and Table 2. (DOCX 16 kb)

Additional file 4: **Table S4.** Genetic correlation (*r*_g_, upper triangular; *p* value, lower triangular) among psychiatric disorders and behavioral traits. *p* values surviving Bonferroni multiple testing correction are reported in *red*. Abbreviations are reported in Table 1 and Table 2. (DOCX 14 kb)

Additional file 5: **Table S5.** Comparisons of regression models including 10 principal components (10PC) vs. 20 principal components (20PC). The 10PC model is the model tested in the main analysis and included as covariates 10 principal components derived from genetic information. The 20PC model was run on the top significant findings and included as covariates 20 principal components derived from genetic information. P10vs20 is the *p* value calculated testing the difference between these two models. Abbreviations are reported in Table 1 and Table 2. (DOCX 13 kb)

Additional file 6: **Table S6.** Best associations for each of the PRS × local-adaptation variables tested. Abbreviations are reported in Table 1 and Table 2. (DOCX 27 kb)

Additional file 7: **Figure S1.** Null distribution generated from 10,000 random permutations of the significant PRS datasets. *Blue lines* represent the observed results. Abbreviations are reported in Table 1 and Table 2. (DOCX 135 kb)

Additional file 8: **Table S7.** Association between height PRS and local-adaptation variables. Abbreviations are reported in Table 2. (DOCX 12 kb)

Additional file 9: **Table S8.** Covariate analysis of SCZ PRS with respect to winter minimum temperature (WMinTemp), winter maximum temperature (WMaxTemp), and longitude (LON). We report top PRS cutoff and cutoff obtained from the main analysis. (DOCX 12 kb)

Additional file 10: **Table S9.** Gene Ontology (GO) enrichment in the SCZ-WinMinTemp (Abbreviations are reported in Table 1 and Table 2) result that survived FDR multiple testing correction (*q* < 0.05). (DOCX 12 kb)

Additional file 11: **Table S10.** Gene Ontology (GO) enrichment in the MDD-altitude result that survived FDR multiple testing correction (*q* < 0.05). Abbreviations are reported in Table 1 and Table 2. (DOCX 14 kb)

Additional file 12: **Figure S2.** Distribution of the results of GO enrichment analysis from 100 random sets. *Orange line* represents *q* < 0.05. (DOCX 225 kb)

Additional file 13: **Figure S3.** Overall distribution of *q* values generated from the GO enrichment analysis of 100 random sets. The *red line* represents *q* < 0.05. (DOCX 69 kb)

Additional file 14: **Figure S4.** Significant association of rs6992714 with GGH gene expression. (DOCX 140 kb)

Additional file 15: **Table S11.** Addictive effects in variants included in single-locus and oligogenic PRS from NOIA analysis. (DOCX 12 kb)

Additional file 16: **Table S12.** Significant associations of rs10932966 with *RP11-16P6.1* gene expression in multiple tissues. (DOCX 11 kb)

## Abbreviations

BD: Bipolar disorder
DS: Depressive symptoms
eQTL: Expression quantitative trait locus
FDR: False discovery rate
GGH: Gamma-glutamyl hydrolase
GIDEON: Global Infectious Diseases and Epidemiology Online Network
GO: Gene Ontology
GWAS: Genome-wide association study
LD: Linkage disequilibrium
MDD: Major depressive disorder
NOIA: Natural and Orthogonal InterAction
OPEN: Openness to experience
PRS: Polygenic risk score
SCZ: Schizophrenia
WinMaxTemp: Winter maximum temperature
WinMinTem: Winter minimum temperature

## References

[1] Polimanti R, Gelernter J (2017). Widespread signatures of positive selection in common risk alleles associated to autism spectrum disorder. PLoS Genet.

[2] Xu K, Schadt EE, Pollard KS, Roussos P, Dudley JT (2015). Genomic and network patterns of schizophrenia genetic variation in human evolutionary accelerated regions. Mol Biol Evol.

[3] Srinivasan S, Bettella F, Mattingsdal M, Wang Y, Witoelar A, Schork AJ, Thompson WK, Zuber V, Winsvold BS, Schizophrenia Working Group of the Psychiatric Genomics Consortium TIHGC (2016). Genetic markers of human evolution are enriched in schizophrenia. Biol Psychiatry.

[4] Beiter ER, Khramtsova EA, van der Merwe C, Chimusa ER, Simonti C, Stein J, Thompson P, Fisher S, Stein DJ, Capra JA (2017). Polygenic selection underlies evolution of human brain structure and behavioral traits. bioRxiv.

[5] Mullins N, Ingason A, Porter H, Euesden J, Gillett A, Olafsson S, Gudbjartsson DF, Lewis CM, Sigurdsson E, Saemundsen E (2017). Reproductive fitness and genetic risk of psychiatric disorders in the general population. Nat Commun.

[6] Daub JT, Hofer T, Cutivet E, Dupanloup I, Quintana-Murci L, Robinson-Rechavi M, Excoffier L (2013). Evidence for polygenic adaptation to pathogens in the human genome. Mol Biol Evol.

[7] Hansen ME, Hunt SC, Stone RC, Horvath K, Herbig U, Ranciaro A, Hirbo J, Beggs W, Reiner AP, Wilson JG (2016). Shorter telomere length in Europeans than in Africans due to polygenetic adaptation. Hum Mol Genet.

[8] Polimanti R, Yang BZ, Zhao H, Gelernter J (2016). Evidence of polygenic adaptation in the systems genetics of anthropometric traits. PLoS One.

[9] Berg JJ, Coop G (2014). A population genetic signal of polygenic adaptation. PLoS Genet.

[10] Hancock AM, Witonsky DB, Gordon AS, Eshel G, Pritchard JK, Coop G, Di Rienzo A (2008). Adaptations to climate in candidate genes for common metabolic disorders. PLoS Genet.

[11] Polimanti R, Piacentini S, Iorio A, De Angelis F, Kozlov A, Novelletto A, Fuciarelli M (2015). Haplotype differences for copy number variants in the 22q11.23 region among human populations: a pigmentation-based model for selective pressure. Eur J Hum Genet.

[12] Turchin MC, Chiang CW, Palmer CD, Sankararaman S, Reich D, Hirschhorn JN, Genetic Investigation of ATC (2012). Evidence of widespread selection on standing variation in Europe at height-associated SNPs. Nat Genet.

[13] Martin AR, Gignoux CR, Walters RK, Wojcik GL, Neale BM, Gravel S, Daly MJ, Bustamante CD, Kenny EE (2017). Human demographic history impacts genetic risk prediction across diverse populations. Am J Hum Genet.

[14] Key FM, Fu Q, Romagne F, Lachmann M, Andres AM (2016). Human adaptation and population differentiation in the light of ancient genomes. Nat Commun.

[15] Psychiatric GWAS Consortium Bipolar Disorder Working Group (2011). Large-scale genome-wide association analysis of bipolar disorder identifies a new susceptibility locus near ODZ4. Nat Genet.

[16] Ripke S, Wray NR, Lewis CM, Hamilton SP, Weissman MM, Breen G, Byrne EM, Blackwood DH, Boomsma DI, Major Depressive Disorder Working Group of the Psychiatric GWAS Consortium (2013). A mega-analysis of genome-wide association studies for major depressive disorder. Mol Psychiatry.

[17] Schizophrenia Working Group of the Psychiatric Genomics Consortium (2014). Biological insights from 108 schizophrenia-associated genetic loci. Nature.

[18] de Moor MH, Costa PT, Terracciano A, Krueger RF, de Geus EJ, Toshiko T, Penninx BW, Esko T, Madden PA, Derringer J (2012). Meta-analysis of genome-wide association studies for personality. Mol Psychiatry.

[19] van den Berg SM, de Moor MH, Verweij KJ, Krueger RF, Luciano M, Arias Vasquez A, Matteson LK, Derringer J, Esko T, Amin N (2016). Meta-analysis of genome-wide association studies for extraversion: findings from the Genetics of Personality Consortium. Behav Genet.

[20] de Moor MH, van den Berg SM, Verweij KJ, Krueger RF, Luciano M, Arias Vasquez A, Matteson LK, Derringer J, Esko T, Genetics of Personality Consortium (2015). Meta-analysis of genome-wide association studies for neuroticism, and the polygenic association with major depressive disorder. JAMA Psychiatry.

[21] Okbay A, Baselmans BM, De Neve JE, Turley P, Nivard MG, Fontana MA, Meddens SF, Linner RK, Rietveld CA, Derringer J (2016). Genetic variants associated with subjective well-being, depressive symptoms, and neuroticism identified through genome-wide analyses. Nat Genet.

[22] Lao O, Lu TT, Nothnagel M, Junge O, Freitag-Wolf S, Caliebe A, Balascakova M, Bertranpetit J, Bindoff LA, Comas D (2008). Correlation between genetic and geographic structure in Europe. Curr Biol.

[23] Delaneau O, Marchini J, Zagury JF (2011). A linear complexity phasing method for thousands of genomes. Nat Methods.

[24] Howie B, Marchini J, Stephens M (2011). Genotype imputation with thousands of genomes. G3 (Bethesda).

[25] Auton A, Brooks LD, Durbin RM, Garrison EP, Kang HM, Korbel JO, Marchini JL, McCarthy S, McVean GA, Abecasis GR, 1000 Genomes Project Consortium (2015). A global reference for human genetic variation. Nature.

[26] Chang CC, Chow CC, Tellier LC, Vattikuti S, Purcell SM, Lee JJ (2015). Second-generation PLINK: rising to the challenge of larger and richer datasets. Gigascience.

[27] Galinsky KJ, Bhatia G, Loh PR, Georgiev S, Mukherjee S, Patterson NJ, Price AL (2016). Fast principal-component analysis reveals convergent evolution of ADH1B in Europe and East Asia. Am J Hum Genet.

[28] Polimanti R, Amstadter AB, Stein MB, Almli LM, Baker DG, Bierut LJ, Bradley B, Farrer LA, Johnson EO, King A (2017). A putative causal relationship between genetically determined female body shape and posttraumatic stress disorder. Genome Med.

[29] Polimanti R, Chen CY, Ursano RJ, Heeringa SG, Jain S, Kessler RC, Nock MK, Smoller JW, Sun X, Gelernter J, Stein MB (2017). Cross-phenotype polygenic risk score analysis of persistent post-concussive symptoms in U.S. Army soldiers with deployment-acquired traumatic brain injury. J Neurotrauma.

[30] Polimanti R, Kaufman J, Zhao H, Kranzler HR, Ursano RJ, Kessler RC, Stein MB, Gelernter J (2018). Trauma exposure interacts with the genetic risk of bipolar disorder in alcohol misuse of US soldiers. Acta Psychiatr Scand.

[31] Zhou H, Polimanti R, Yang BZ, Wang Q, Han S, Sherva R, Nunez YZ, Zhao H, Farrer LA, Kranzler HR, Gelernter J (2017). Genetic risk variants associated with comorbid alcohol dependence and major depression. JAMA Psychiatry.

[32] Wang Q, Polimanti R, Kranzler HR, Farrer LA, Zhao H, Gelernter J (2017). Genetic factor common to schizophrenia and HIV infection is associated with risky sexual behavior: antagonistic vs. synergistic pleiotropic SNPs enriched for distinctly different biological functions. Hum Genet.

[33] Moran S, McCloy D, Wright R (2014). PHOIBLE Online.

[34] Euesden J, Lewis CM, O'Reilly PF (2015). PRSice: polygenic risk score software. Bioinformatics.

[35] Benjamini Y, Hochberg Y (1995). Controlling the false discovery rate: a practical and powerful approach to multiple testing. J R Stat Soc Ser B Methodol.

[36] Zheng J, Erzurumluoglu AM, Elsworth BL, Kemp JP, Howe L, Haycock PC, Hemani G, Tansey K, Laurin C, Early G (2017). LD Hub: a centralized database and web interface to perform LD score regression that maximizes the potential of summary level GWAS data for SNP heritability and genetic correlation analysis. Bioinformatics.

[37] Bulik-Sullivan B, Finucane HK, Anttila V, Gusev A, Day FR, Loh PR, ReproGen C, Psychiatric Genomics C, Duncan L, Genetic Consortium for Anorexia Nervosa of the Wellcome Trust Case Control C (2015). An atlas of genetic correlations across human diseases and traits. Nat Genet.

[38] Li J, Wang L, Jiang T, Wang J, Li X, Liu X, Wang C, Teng Z, Zhang R, Lv H, Guo M (2016). eSNPO: An eQTL-based SNP ontology and SNP functional enrichment analysis platform. Sci Rep.

[39] Pers TH, Timshel P, Hirschhorn JN (2015). SNPsnap: a Web-based tool for identification and annotation of matched SNPs. Bioinformatics.

[40] Alvarez-Castro JM, Carlborg O (2007). A unified model for functional and statistical epistasis and its application in quantitative trait loci analysis. Genetics.

[41] Harris RA, Alcott CE, Sullivan EL, Takahashi D, McCurdy CE, Comstock S, Baquero K, Blundell P, Frias AE, Kahr M (2016). Genomic variants associated with resistance to high fat diet induced obesity in a primate model. Sci Rep.

[42] GTEx Consortium (2013). The Genotype-Tissue Expression (GTEx) project. Nat Genet.

[43] Pybus M, Luisi P, Dall'Olio GM, Uzkudun M, Laayouni H, Bertranpetit J, Engelken J (2015). Hierarchical boosting: a machine-learning framework to detect and classify hard selective sweeps in human populations. Bioinformatics.

[44] Field Y, Boyle EA, Telis N, Gao Z, Gaulton KJ, Golan D, Yengo L, Rocheleau G, Froguel P, McCarthy MI, Pritchard JK (2016). Detection of human adaptation during the past 2000 years. Science.

[45] Mortensen PB, Pedersen CB, Westergaard T, Wohlfahrt J, Ewald H, Mors O, Andersen PK, Melbye M (1999). Effects of family history and place and season of birth on the risk of schizophrenia. N Engl J Med.

[46] Racimo F, Gokhman D, Fumagalli M, Ko A, Hansen T, Moltke I, Albrechtsen A, Carmel L, Huerta-Sanchez E, Nielsen R (2017). Archaic adaptive introgression in TBX15/WARS2. Mol Biol Evol.

[47] Okerlund ND, Cheyette BN (2011). Synaptic Wnt signaling—a contributor to major psychiatric disorders?. J Neurodev Disord.

[48] Holstein TW (2012). The evolution of the Wnt pathway. Cold Spring Harb Perspect Biol.

[49] Bauer M, Glenn T, Alda M, Andreassen OA, Angelopoulos E, Ardau R, Baethge C, Bauer R, Baune BT, Bellivier F (2015). Influence of light exposure during early life on the age of onset of bipolar disorder. J Psychiatr Res.

[50] Dauphinais DR, Rosenthal JZ, Terman M, DiFebo HM, Tuggle C, Rosenthal NE (2012). Controlled trial of safety and efficacy of bright light therapy vs. negative air ions in patients with bipolar depression. Psychiatry Res.

[51] Cohen JM, Civitello DJ, Brace AJ, Feichtinger EM, Ortega CN, Richardson JC, Sauer EL, Liu X, Rohr JR (2016). Spatial scale modulates the strength of ecological processes driving disease distributions. Proc Natl Acad Sci U S A.

[52] Xie JL, Grahl N, Sless T, Leach MD, Kim SH, Hogan DA, Robbins N, Cowen LE (2016). Signaling through Lrg1, Rho1 and Pkc1 governs Candida albicans morphogenesis in response to diverse cues. PLoS Genet.

[53] Tomkins A (1981). Tropical malabsorption: recent concepts in pathogenesis and nutritional significance. Clin Sci (Lond).

[54] Creanza N, Ruhlen M, Pemberton TJ, Rosenberg NA, Feldman MW, Ramachandran S (2015). A comparison of worldwide phonemic and genetic variation in human populations. Proc Natl Acad Sci U S A.

[55] Moyer A (2014). Exceptional outcomes in L2 phonology: the critical factors of learner engagement and self-regulation. Appl Linguist.

