# Supplementary material for: Local adaptation in European populations affected the genetics of psychiatric disorders and behavioral traits
Source: Genome Med. 2018 Mar 26;10:24. doi: 10.1186/s13073-018-0532-7 (PMC5870256; doi:10.1186/s13073-018-0532-7)
Supplement: Supplementary file 1 — Table S1. Details of the European samples investigated. (DOCX 12 kb) [file 13073_2018_532_MOESM1_ESM.docx]

**Additional file 1: Table S1 -** Details of the European samples investigated.

| **Country** | **City** | **Sample size** |
| --- | --- | --- |
| Austria | Innsbruck | 50 |
| Switzerland | Lausanne | 133 |
| Czech Republic | Prague | 45 |
| Germany (Northern) | Kiel | 494 |
| Germany (Southern) | Augsburg | 488 |
| Denmark | Kopenhagen | 59 |
| Greece (Northern) | - | 51 |
| Spain (Central) | Madrid | 81 |
| Spain (Eastern) | Barcelona | 47 |
| Finland | Helsinki | 47 |
| France | Lyon | 50 |
| Hungary | Budapest | 17 |
| Ireland | Dublin | 35 |
| Italy (Central-Western) | Rome | 106 |
| Italy (Central-Eastern) | Ancona | 49 |
| Netherlands | Rotterdam | 280 |
| Norway | Førde | 52 |
| Poland | Warsawa | 49 |
| Portugal | Lisboa | 16 |
| Romania | Bucharest | 12 |
| Serbia | Belgrade | 55 |
| Sweden | Uppsala | 45 |
| United Kingdom | London | 194 |
