# Supplementary material for: Local adaptation in European populations affected the genetics of psychiatric disorders and behavioral traits
Source: Genome Med. 2018 Mar 26;10:24. doi: 10.1186/s13073-018-0532-7 (PMC5870256; doi:10.1186/s13073-018-0532-7)
Supplement: Supplementary file 2 — Table S2. Heritability statistics (LD score regression) of the GWASs considered. (DOCX 12 kb) [file 13073_2018_532_MOESM2_ESM.docx]

**Additional file 2: Table S2 -** Heritability statistics (LD score regression) of the GWAS considered.

| **GWAS** | **Z_h2** | **Lambda GC** | **Mean Chi^2** |
| --- | --- | --- | --- |
| Agreeableness | 0.56 | 1.00 | 1.01 |
| Autism Spectrum Disorder | 9.05 | 1.05 | 1.06 |
| Bipolar Disorder | 11.71 | 1.15 | 1.16 |
| Conscientiousness | 2.45 | 1.02 | 1.03 |
| Depressive Symptoms | 12.86 | 1.13 | 1.15 |
| Extraversion | 5.62 | 1.06 | 1.07 |
| Major Depressive Disorder | 6.74 | 1.06 | 1.07 |
| Neuroticism (GPC) | 4.55 | 1.05 | 1.06 |
| Neuroticism (SSGAC) | 12.96 | 1.24 | 1.31 |
| Openness to Experience | 3.97 | 1.03 | 1.03 |
| Schizophrenia | 23.17 | 1.60 | 1.81 |
| Subjective Well-Being | 11.95 | 1.13 | 1.15 |
