# Supplementary material for: Local adaptation in European populations affected the genetics of psychiatric disorders and behavioral traits
Source: Genome Med. 2018 Mar 26;10:24. doi: 10.1186/s13073-018-0532-7 (PMC5870256; doi:10.1186/s13073-018-0532-7)
Supplement: Supplementary file 3 — Table S3. Correlations (Spearman’s rho, upper triangular; p value, lower triangular) among variables related to local adaptation. p values surviving Bonferroni multiple testing correction are reported in red. Abbreviations are reported in Table 1 and Table 2. (DOCX 16 kb) [file 13073_2018_532_MOESM3_ESM.docx]

Additional file 3: Table S3 - Correlations (Spearman’s rho, upper triangular; p value, lower triangular) among variables related to local adaptation. P value surviving Bonferroni multiple testing correction are reported in red. Abbreviations are reported in Table 1 and Table 2.

|  | **LAT** | **LON** | **ALT** | **SMaT** | **SMiT** | **WMaT** | **WMiT** | **MaPR** | **MiPR** | **MaRH** | **MiRH** | **MaSD** | **MiSDa** | **Virus** | **Bacteria** | **Protozoa** | **Segments** | **Vowels** | **Consonants** |
| --- | --- | --- | --- | --- | --- | --- | --- | --- | --- | --- | --- | --- | --- | --- | --- | --- | --- | --- | --- |
| **LAT** |  | 0.04 | -0.27 | **-0.93** | **-0.77** | -0.68 | -0.46 | -0.02 | 0.55 | **0.73** | 0.67 | **-0.86** | **-0.91** | -0.31 | **-0.74** | **-0.80** | 0.51 | 0.58 | 0.21 |
| **LON** | 8.68E-01 |  | 0.08 | 0.21 | 0.12 | -0.47 | -0.60 | -0.07 | -0.17 | 0.09 | -0.40 | 0.29 | -0.08 | 0.44 | -0.21 | -0.05 | -0.41 | -0.33 | -0.32 |
| **ALT** | 2.09E-01 | 7.03E-01 |  | 0.29 | -0.10 | -0.18 | -0.35 | 0.08 | -0.06 | -0.15 | -0.28 | 0.27 | 0.26 | 0.35 | 0.01 | 0.38 | -0.07 | 0.01 | -0.15 |
| **SMaT** | **1.06E-10** | 3.28E-01 | 1.73E-01 |  | **0.76** | 0.50 | 0.21 | -0.10 | -0.64 | -0.68 | **-0.76** | **0.92** | **0.82** | 0.42 | 0.64 | **0.76** | -0.64 | -0.70 | -0.34 |
| **SMiT** | **1.63E-05** | 5.77E-01 | 6.51E-01 | **2.22E-05** |  | 0.61 | 0.46 | -0.07 | -0.59 | -0.67 | -0.66 | 0.69 | **0.72** | 0.22 | 0.66 | **0.75** | -0.40 | -0.55 | -0.04 |
| **WMaT** | 3.21E-04 | 2.42E-02 | 4.24E-01 | 1.62E-02 | 2.15E-03 |  | **0.90** | 0.15 | -0.31 | -0.60 | -0.20 | 0.46 | 0.69 | 0.02 | **0.72** | 0.62 | -0.16 | -0.22 | 0.04 |
| **WMiT** | 2.81E-02 | 2.26E-03 | 1.01E-01 | 3.26E-01 | 2.65E-02 | **7.08E-09** |  | 0.08 | -0.21 | -0.41 | 0.02 | 0.25 | 0.45 | -0.13 | 0.59 | 0.42 | -0.02 | -0.13 | 0.22 |
| **MaPR** | 9.29E-01 | 7.38E-01 | 7.14E-01 | 6.50E-01 | 7.37E-01 | 4.96E-01 | 7.32E-01 |  | 0.45 | -0.24 | 0.24 | -0.09 | 0.11 | 0.05 | 0.01 | -0.05 | 0.15 | 0.32 | 0.16 |
| **MiPR** | 6.16E-03 | 4.30E-01 | 7.86E-01 | 9.17E-04 | 2.81E-03 | 1.51E-01 | 3.44E-01 | 3.31E-02 |  | 0.25 | 0.45 | -0.65 | -0.48 | -0.30 | -0.58 | -0.62 | 0.61 | 0.65 | 0.24 |
| **MaRH** | **7.10E-05** | 6.89E-01 | 4.97E-01 | 3.50E-04 | 5.35E-04 | 2.66E-03 | 5.47E-02 | 2.67E-01 | 2.56E-01 |  | 0.54 | -0.57 | **-0.80** | 0.03 | -0.45 | -0.65 | 0.28 | 0.39 | 0.02 |
| **MiRH** | 5.27E-04 | 5.96E-02 | 2.02E-01 | **3.03E-05** | 6.38E-04 | 3.54E-01 | 9.15E-01 | 2.72E-01 | 3.31E-02 | 7.41E-03 |  | **-0.75** | -0.57 | -0.26 | -0.35 | -0.51 | 0.60 | 0.57 | 0.55 |
| **MaSD** | **1.55E-07** | 1.87E-01 | 2.15E-01 | **5.41E-10** | 2.43E-04 | 2.64E-02 | 2.57E-01 | 6.98E-01 | 7.25E-04 | 4.15E-03 | **3.37E-05** |  | **0.74** | 0.51 | 0.62 | 0.68 | -0.72 | -0.65 | -0.49 |
| **MiSD** | **1.74E-09** | 7.33E-01 | 2.25E-01 | **1.51E-06** | **9.82E-05** | 2.97E-04 | 3.12E-02 | 6.18E-01 | 2.07E-02 | **5.72E-06** | 4.42E-03 | **5.26E-05** |  | 0.16 | 0.57 | **0.78** | -0.40 | -0.48 | -0.05 |
| **Virus** | 1.55E-01 | 3.50E-02 | 9.67E-02 | 4.69E-02 | 3.20E-01 | 9.30E-01 | 5.56E-01 | 8.32E-01 | 1.68E-01 | 8.82E-01 | 2.36E-01 | 1.26E-02 | 4.67E-01 |  | 0.45 | 0.41 | -0.50 | -0.40 | -0.32 |
| **Bacteria** | **6.17E-05** | 3.29E-01 | 9.67E-01 | 8.98E-04 | 6.13E-04 | **1.18E-04** | 2.92E-03 | 9.54E-01 | 3.47E-03 | 2.98E-02 | 9.86E-02 | 1.65E-03 | 4.20E-03 | 3.26E-02 |  | 0.71 | -0.45 | -0.50 | -0.14 |
| **Protozoa** | **5.54E-06** | 8.24E-01 | 7.66E-02 | **2.48E-05** | **4.51E-05** | 1.71E-03 | 4.80E-02 | 8.25E-01 | 1.49E-03 | 7.50E-04 | 1.20E-02 | 3.20E-04 | **1.04E-05** | 4.94E-02 | 1.69E-04 |  | -0.40 | -0.48 | -0.07 |
| **Segments** | 1.61E-02 | 5.53E-02 | 7.69E-01 | 1.31E-03 | 6.88E-02 | 4.64E-01 | 9.30E-01 | 4.93E-01 | 2.42E-03 | 2.07E-01 | 2.88E-03 | 1.71E-04 | 6.66E-02 | 1.91E-02 | 3.60E-02 | 6.88E-02 |  | **0.83** | 0.62 |
| **Vowels** | 4.51E-03 | 1.28E-01 | 9.74E-01 | 2.65E-04 | 7.55E-03 | 3.33E-01 | 5.71E-01 | 1.50E-01 | 1.16E-03 | 6.96E-02 | 5.70E-03 | 1.12E-03 | 2.42E-02 | 6.74E-02 | 1.83E-02 | 2.23E-02 | **1.73E-06** |  | 0.20 |
| **Consonants** | 3.45E-01 | 1.53E-01 | 5.14E-01 | 1.18E-01 | 8.68E-01 | 8.56E-01 | 3.15E-01 | 4.68E-01 | 2.89E-01 | 9.38E-01 | 8.48E-03 | 1.95E-02 | 8.18E-01 | 1.47E-01 | 5.25E-01 | 7.55E-01 | 1.96E-03 | 3.72E-01 |  |
