# Supplementary material for: Local adaptation in European populations affected the genetics of psychiatric disorders and behavioral traits
Source: Genome Med. 2018 Mar 26;10:24. doi: 10.1186/s13073-018-0532-7 (PMC5870256; doi:10.1186/s13073-018-0532-7)
Supplement: Supplementary file 4 — Table S4. Genetic correlation (rg, upper triangular; p value, lower triangular) among psychiatric disorders and behavioral traits. p values surviving Bonferroni multiple testing correction are reported in red. Abbreviations are reported in Table 1 and Table 2. (DOCX 14 kb) [file 13073_2018_532_MOESM4_ESM.docx]

Additional file 4: Table S4 - Genetic correlation (r_g_, upper triangular; p value, lower triangular) among psychiatric disorders and behavioral traits. P value surviving Bonferroni multiple testing correction are reported in red. Abbreviations are reported in Table 1 and Table 2.

|  | **DS** | **CONS** | **OPEN** | **gpcNEURO** | **ssgacNEURO** | **ASD** | **BD** | **MDD** | **SCZ** | **SWB** | **EXTRA** | **AGREE** |
| --- | --- | --- | --- | --- | --- | --- | --- | --- | --- | --- | --- | --- |
| **DS** |  | -0.34 | -0.07 | **0.98** | **0.72** | 0.11 | **0.25** | **1.00** | **0.31** | **-0.78** | -0.23 | -1.00 |
| **CONS** | 1.10E-02 |  | 0.29 | -0.80 | -0.36 | -0.26 | -0.18 | -0.46 | -0.10 | 0.50 | 0.48 | 0.23 |
| **OPEN** | 5.05E-01 | 2.65E-01 |  | 0.08 | 0.02 | 0.42 | 0.19 | 0.10 | 0.19 | 0.04 | 0.37 | 0.91 |
| **gpcNEURO** | **9.60E-14** | 6.00E-03 | 6.88E-01 |  | **1.00** | 0.08 | 0.14 | **1.00** | 0.18 | **-1.00** | -0.17 | -1.00 |
| **ssgacNEURO** | **9.93E-145** | 3.00E-03 | 8.46E-01 | **1.22E-20** |  | 0.15 | 0.11 | **0.78** | **0.20** | **-0.74** | **-0.49** | -0.92 |
| **ASD** | 1.45E-01 | 1.44E-01 | 2.00E-03 | 4.84E-01 | 8.00E-02 |  | 0.06 | 0.14 | **0.18** | **-0.26** | -0.22 | -0.09 |
| **BD** | **1.89E-06** | 1.99E-01 | 6.40E-02 | 8.90E-02 | 1.10E-02 | 4.12E-01 |  | **0.64** | **0.83** | **-0.20** | 0.24 | -0.17 |
| **MDD** | **1.77E-36** | 1.60E-02 | 4.78E-01 | **4.79E-10** | **5.16E-18** | 1.76E-01 | **3.01E-15** |  | **0.46** | **-0.82** | -0.26 | -0.30 |
| **SCZ** | **6.01E-15** | 2.55E-01 | 1.00E-02 | 7.00E-03 | **2.97E-07** | **1.93E-04** | **5.31E-106** | **1.09E-14** |  | **-0.30** | -0.02 | -0.40 |
| **SWB** | **5.42E-48** | 2.00E-03 | 7.35E-01 | **2.05E-13** | **2.70E-77** | **7.06E-05** | **2.33E-04** | **3.15E-13** | **1.60E-13** |  | **0.64** | 1.00 |
| **EXTRA** | 7.40E-03 | 2.80E-02 | 1.22E-02 | 1.84E-01 | **7.08E-12** | 2.65E-02 | 7.50E-03 | 7.71E-02 | 6.90E-01 | **4.74E-13** |  | -0.27 |
| **AGREE** | 4.26E-01 | 6.70E-01 | 4.10E-01 | 4.73E-01 | 4.18E-01 | 8.03E-01 | 6.53E-01 | 5.11E-01 | 2.13E-01 | 3.79E-01 | 6.46E-01 |  |
