# Supplementary material for: Local adaptation in European populations affected the genetics of psychiatric disorders and behavioral traits
Source: Genome Med. 2018 Mar 26;10:24. doi: 10.1186/s13073-018-0532-7 (PMC5870256; doi:10.1186/s13073-018-0532-7)
Supplement: Supplementary file 5 — Table S5. Comparisons of regression models including 10 principal components (10PC) vs. 20 principal components (20PC). The 10PC model is the model tested in the main analysis and included as covariates 10 principal components derived from genetic information. The 20PC model was run on the top significant findings and included as covariates 20 principal components derived from genetic information. P10vs20 is the p value calculated testing the difference between these two models. Abbreviations are reported in Table 1 and Table 2. (DOCX 13 kb) [file 13073_2018_532_MOESM5_ESM.docx]

Additional file 5: Table S5 - Comparisons of regression models including 10 principal components (10PC) vs. 20 principal components (20PC). The 10PC model is the model tested in the main analysis and included as covariates 10 principal components derived from genetic information. The 20PC model was run on the top significant findings and included as covariates 20 principal components derived from genetic information. The P_10vs20_ is the p value calculated testing the difference between these two models. Abbreviations are reported in Table 1 and Table 2.

| **Local-Adaptation variable** | **PRS** | **PT** | **SNP N** | **Z_10PC_** | **Z_20PC_** | **P_10vs20_** |
| --- | --- | --- | --- | --- | --- | --- |
| MaxSunnyDaylight | BD | 0.01 | 2,833 | -2.93 | -2.74 | 0.831 |
| Consonants | CONS | 0.5 | 60,620 | -2.97 | -2.90 | 0.987 |
| Latitude | DS | 10^-7^ | 1 | 3.47 | 3.22 | 0.782 |
| SumMaxTemp |  |  |  | -3.40 | -3.13 | 0.788 |
| Min PrecipRate |  | 0.05 | 12,832 | -3.29 | -3.10 | 0.747 |
| MaxPrecipRate | MDD | 0.3 | 39,390 | -3.21 | -3.16 | 0.752 |
| Altitude |  | 1 | 97,481 | -3.13 | -3.50 | 0.777 |
| ProtozoaDiversity | OPEN | 10^-6^ | 2 | 3.56 | 3.77 | 0.869 |
| SumMinTemp |  | 5x10^-8^ | 1 | 2.7 | 2.66 | 0.839 |
| WinMinTemp | SCZ | 0.5 | 104,106 | 3.84 | 3.89 | 0.942 |
| Longitude |  |  |  | -3.29 | -2.87 | 0.799 |
| WinMaxTemp |  |  |  | 2.96 | 3.32 | 0.802 |
| MinRelHumidity | SWB | 10^-6^ | 4 | -2.95 | -2.61 | 0.769 |
