# Supplementary material for: Local adaptation in European populations affected the genetics of psychiatric disorders and behavioral traits
Source: Genome Med. 2018 Mar 26;10:24. doi: 10.1186/s13073-018-0532-7 (PMC5870256; doi:10.1186/s13073-018-0532-7)
Supplement: Supplementary file 6 — Table S6. Best associations for each of the PRS × local-adaptation variables tested. Abbreviations are reported in Table 1 and Table 2. (DOCX 27 kb) [file 13073_2018_532_MOESM6_ESM.docx]

**Additional file 6: Table S6 -** Best associations for each of the PRS x local-adaptation variables tested. Abbreviations are reported in Table 1 and Table 2.

| **Local-Adaptation variable** | **PRS** | **PT** | **SNP N** | **Z score** | **P value** | **Q value** |
| --- | --- | --- | --- | --- | --- | --- |
| WinMinTemp | SCZ | 0.5 | 104106 | 3.84 | 1.28E-04 | 0.029 |
| ProtozoaDiversity | OPEN | 1.00E-06 | 2 | 3.56 | 3.82E-04 | 0.029 |
| LAT | DS | 1.00E-07 | 1 | 3.47 | 5.38E-04 | 0.029 |
| SumMaxTemp | DS | 1.00E-07 | 1 | -3.40 | 6.91E-04 | 0.029 |
| LAT | CONS | 1.00E-05 | 3 | 3.36 | 7.92E-04 | 0.029 |
| WinMinTemp | MDD | 0.05 | 8160 | 3.34 | 8.46E-04 | 0.029 |
| LON | SCZ | 0.5 | 104106 | -3.29 | 1.01E-03 | 0.029 |
| MINPrecipRate | DS | 0.05 | 12832 | -3.29 | 1.03E-03 | 0.029 |
| MaxPrecipRate | MDD | 0.3 | 39390 | -3.21 | 1.33E-03 | 0.034 |
| WinMinTemp | EXTRA | 1.00E-05 | 7 | 3.14 | 1.74E-03 | 0.037 |
| ALT | MDD | 1 | 97481 | -3.13 | 1.79E-03 | 0.037 |
| LAT | AGREE | 0.001 | 232 | 2.99 | 2.81E-03 | 0.043 |
| MaxPrecipRate | SWB | 1.00E-06 | 4 | -2.99 | 2.84E-03 | 0.043 |
| Consonants | CONS | 0.5 | 60620 | -2.97 | 2.98E-03 | 0.043 |
| SumMinTemp | OPEN | 5.00E-08 | 1 | 2.97 | 3.02E-03 | 0.043 |
| WinMaxTemp | SCZ | 0.5 | 104106 | 2.96 | 3.09E-03 | 0.043 |
| MinRelHumidity | SWB | 1.00E-06 | 4 | -2.95 | 3.22E-03 | 0.043 |
| MaxSunnyDaylight | BD | 0.01 | 2833 | -2.93 | 3.42E-03 | 0.043 |
| Consonants | EXTRA | 0.01 | 3261 | 2.87 | 4.13E-03 | 0.049 |
| LON | EXTRA | 0.001 | 436 | -2.86 | 4.33E-03 | 0.049 |
| ALT | SCZ | 0.5 | 104106 | -2.79 | 5.35E-03 | 0.056 |
| MaxRelHumidity | SWB | 0.01 | 2698 | 2.78 | 5.48E-03 | 0.056 |
| VirusDiversity | MDD | 0.001 | 223 | -2.77 | 5.69E-03 | 0.056 |
| Consonants | SCZ | 0.5 | 104106 | 2.75 | 6.04E-03 | 0.057 |
| LON | MDD | 1 | 97481 | -2.74 | 6.24E-03 | 0.057 |
| MinSunnyDaylight | AGREE | 0.001 | 232 | -2.72 | 6.58E-03 | 0.058 |
| ProtozoaDiversity | EXTRA | 0.1 | 22639 | 2.66 | 7.96E-03 | 0.067 |
| MaxPrecipRate | SCZ | 0.1 | 36033 | -2.64 | 8.40E-03 | 0.068 |
| SumMinTemp | DS | 0.01 | 3628 | 2.58 | 9.80E-03 | 0.072 |
| SumMinTemp | EXTRA | 1.00E-04 | 50 | -2.58 | 9.95E-03 | 0.072 |
| Segments | EXTRA | 0.01 | 3261 | 2.57 | 1.01E-02 | 0.072 |
| WinMaxTemp | EXTRA | 1.00E-05 | 7 | 2.54 | 1.10E-02 | 0.072 |
| SumMinTemp | SWB | 1.00E-06 | 4 | 2.54 | 1.11E-02 | 0.072 |
| WinMaxTemp | SWB | 0.1 | 17558 | -2.54 | 1.13E-02 | 0.072 |
| ProtozoaDiversity | MDD | 0.001 | 223 | -2.53 | 1.14E-02 | 0.072 |
| WinMinTemp | OPEN | 5.00E-08 | 1 | 2.53 | 1.14E-02 | 0.072 |
| WinMinTemp | AGREE | 0.01 | 1956 | 2.51 | 1.21E-02 | 0.073 |
| Segments | BD | 0.01 | 2833 | 2.51 | 1.22E-02 | 0.073 |
| BacteriaDiversity | BD | 1.00E-04 | 82 | 2.45 | 1.42E-02 | 0.083 |
| WinMaxTemp | MDD | 0.001 | 223 | -2.44 | 1.49E-02 | 0.085 |
| MaxPrecipRate | EXTRA | 1.00E-05 | 7 | -2.41 | 1.61E-02 | 0.090 |
| Segments | CONS | 0.1 | 15491 | -2.40 | 1.67E-02 | 0.090 |
| ALT | DS | 0.01 | 3628 | -2.37 | 1.77E-02 | 0.094 |
| SumMinTemp | BD | 0.3 | 45899 | 2.36 | 1.86E-02 | 0.095 |
| SumMaxTemp | NEUROgpc | 5.00E-08 | 1 | -2.35 | 1.87E-02 | 0.095 |
| WinMinTemp | DS | 1.00E-05 | 27 | 2.34 | 1.92E-02 | 0.095 |
| MaxRelHumidity | NEUROgpc | 1 | 110359 | -2.32 | 2.03E-02 | 0.097 |
| MinRelHumidity | MDD | 0.001 | 223 | -2.32 | 2.04E-02 | 0.097 |
| MaxPrecipRate | DS | 0.05 | 12832 | -2.28 | 2.24E-02 | 0.102 |
| Vowels | SCZ | 1 | 145943 | -2.28 | 2.25E-02 | 0.102 |
| MaxSunnyDaylight | EXTRA | 0.01 | 3261 | -2.28 | 2.27E-02 | 0.102 |
| ALT | BD | 0.3 | 45899 | -2.26 | 2.41E-02 | 0.105 |
| WinMaxTemp | DS | 1.00E-07 | 1 | 2.25 | 2.45E-02 | 0.105 |
| MaxPrecipRate | OPEN | 5.00E-08 | 1 | -2.22 | 2.64E-02 | 0.108 |
| MinRelHumidity | EXTRA | 1.00E-04 | 50 | 2.22 | 2.64E-02 | 0.108 |
| ALT | AGREE | 0.001 | 232 | -2.21 | 2.70E-02 | 0.108 |
| SumMaxTemp | ASD | 0.001 | 402 | -2.21 | 2.73E-02 | 0.108 |
| Vowels | SWB | 1.00E-06 | 4 | -2.20 | 2.81E-02 | 0.108 |
| SumMaxTemp | CONS | 1.00E-05 | 3 | -2.19 | 2.88E-02 | 0.108 |
| Segments | DS | 0.05 | 12832 | -2.19 | 2.88E-02 | 0.108 |
| ProtozoaDiversity | CONS | 1.00E-04 | 21 | -2.19 | 2.88E-02 | 0.108 |
| Vowels | OPEN | 5.00E-08 | 1 | -2.16 | 3.11E-02 | 0.114 |
| Consonants | MDD | 1 | 97481 | 2.15 | 3.16E-02 | 0.114 |
| VirusDiversity | SWB | 0.3 | 42226 | 2.14 | 3.27E-02 | 0.116 |
| MinRelHumidity | OPEN | 0.1 | 15452 | -2.13 | 3.29E-02 | 0.116 |
| MaxRelHumidity | NEUROssgac | 1.00E-05 | 92 | 2.12 | 3.41E-02 | 0.116 |
| SumMinTemp | SCZ | 0.5 | 104106 | 2.11 | 3.46E-02 | 0.116 |
| Vowels | DS | 0.05 | 12832 | -2.11 | 3.47E-02 | 0.116 |
| MinRelHumidity | DS | 1.00E-06 | 7 | 2.10 | 3.61E-02 | 0.116 |
| MaxPrecipRate | BD | 0.3 | 45899 | -2.09 | 3.66E-02 | 0.116 |
| Segments | SWB | 1.00E-06 | 4 | -2.09 | 3.66E-02 | 0.116 |
| VirusDiversity | BD | 1.00E-05 | 13 | 2.08 | 3.74E-02 | 0.116 |
| MaxSunnyDaylight | SWB | 0.001 | 453 | -2.08 | 3.75E-02 | 0.116 |
| MINPrecipRate | OPEN | 5.00E-08 | 1 | -2.07 | 3.86E-02 | 0.116 |
| WinMinTemp | SWB | 1 | 90911 | -2.07 | 3.90E-02 | 0.116 |
| MaxRelHumidity | DS | 1.00E-05 | 27 | 2.05 | 4.05E-02 | 0.116 |
| BacteriaDiversity | SWB | 1.00E-06 | 4 | 2.05 | 4.07E-02 | 0.116 |
| MinSunnyDaylight | SCZ | 0.01 | 8546 | 2.04 | 4.10E-02 | 0.116 |
| Consonants | OPEN | 5.00E-08 | 1 | 2.04 | 4.13E-02 | 0.116 |
| Consonants | BD | 0.01 | 2833 | 2.04 | 4.13E-02 | 0.116 |
| WinMinTemp | BD | 0.3 | 45899 | 2.04 | 4.19E-02 | 0.116 |
| MinSunnyDaylight | CONS | 1.00E-05 | 3 | -2.04 | 4.19E-02 | 0.116 |
| WinMaxTemp | AGREE | 0.05 | 8382 | 2.02 | 4.33E-02 | 0.119 |
| WinMinTemp | NEUROssgac | 1 | 103553 | -2.01 | 4.45E-02 | 0.120 |
| BacteriaDiversity | OPEN | 1 | 93105 | -2.01 | 4.49E-02 | 0.120 |
| SumMaxTemp | AGREE | 0.1 | 15420 | -1.99 | 4.73E-02 | 0.125 |
| MaxRelHumidity | EXTRA | 1 | 110345 | 1.97 | 4.86E-02 | 0.127 |
| MINPrecipRate | NEUROgpc | 5.00E-08 | 1 | 1.97 | 4.94E-02 | 0.128 |
| ProtozoaDiversity | DS | 1.00E-07 | 1 | 1.95 | 5.12E-02 | 0.128 |
| Vowels | EXTRA | 1.00E-04 | 50 | 1.95 | 5.16E-02 | 0.128 |
| VirusDiversity | SCZ | 0.5 | 104106 | -1.94 | 5.21E-02 | 0.128 |
| Consonants | SWB | 1.00E-06 | 4 | -1.94 | 5.23E-02 | 0.128 |
| Vowels | ASD | 0.01 | 3305 | 1.93 | 5.35E-02 | 0.128 |
| BacteriaDiversity | EXTRA | 1.00E-05 | 7 | 1.93 | 5.38E-02 | 0.128 |
| ALT | EXTRA | 1.00E-05 | 7 | -1.92 | 5.44E-02 | 0.128 |
| MaxSunnyDaylight | SCZ | 0.5 | 104106 | -1.92 | 5.47E-02 | 0.128 |
| MinRelHumidity | CONS | 0.3 | 40010 | -1.92 | 5.54E-02 | 0.128 |
| MinSunnyDaylight | BD | 0.1 | 18919 | -1.92 | 5.55E-02 | 0.128 |
| Segments | NEUROgpc | 1.00E-04 | 71 | 1.92 | 5.56E-02 | 0.128 |
| WinMaxTemp | NEUROssgac | 1 | 103553 | -1.91 | 5.66E-02 | 0.129 |
| ALT | SWB | 0.3 | 42226 | 1.90 | 5.70E-02 | 0.129 |
| SumMaxTemp | SCZ | 0.1 | 36033 | -1.89 | 5.86E-02 | 0.130 |
| ProtozoaDiversity | ASD | 5.00E-08 | 1 | 1.89 | 5.86E-02 | 0.130 |
| MaxRelHumidity | AGREE | 0.001 | 232 | 1.88 | 6.00E-02 | 0.132 |
| BacteriaDiversity | MDD | 0.001 | 223 | -1.87 | 6.11E-02 | 0.133 |
| MinRelHumidity | BD | 1.00E-05 | 13 | 1.87 | 6.17E-02 | 0.133 |
| VirusDiversity | EXTRA | 1.00E-06 | 2 | 1.86 | 6.31E-02 | 0.134 |
| MaxSunnyDaylight | ASD | 0.001 | 402 | -1.85 | 6.49E-02 | 0.137 |
| MaxRelHumidity | ASD | 0.001 | 402 | 1.82 | 6.86E-02 | 0.144 |
| MINPrecipRate | SWB | 1.00E-06 | 4 | -1.82 | 6.93E-02 | 0.144 |
| VirusDiversity | DS | 0.05 | 12832 | -1.79 | 7.42E-02 | 0.150 |
| ALT | OPEN | 5.00E-08 | 1 | -1.79 | 7.43E-02 | 0.150 |
| LAT | EXTRA | 1.00E-04 | 50 | -1.78 | 7.45E-02 | 0.150 |
| MaxSunnyDaylight | MDD | 1 | 97481 | -1.78 | 7.56E-02 | 0.151 |
| BacteriaDiversity | SCZ | 5.00E-08 | 120 | -1.76 | 7.92E-02 | 0.157 |
| Vowels | BD | 1.00E-05 | 13 | 1.75 | 7.99E-02 | 0.157 |
| LAT | OPEN | 1.00E-04 | 26 | 1.75 | 8.11E-02 | 0.158 |
| LAT | NEUROssgac | 0.001 | 903 | 1.74 | 8.16E-02 | 0.158 |
| LAT | BD | 0.01 | 2833 | 1.73 | 8.36E-02 | 0.160 |
| Consonants | NEUROssgac | 1.00E-06 | 43 | 1.73 | 8.43E-02 | 0.160 |
| SumMaxTemp | BD | 0.01 | 2833 | -1.71 | 8.67E-02 | 0.163 |
| MaxPrecipRate | ASD | 0.1 | 25330 | 1.71 | 8.83E-02 | 0.165 |
| MinRelHumidity | ASD | 0.001 | 402 | 1.69 | 9.17E-02 | 0.168 |
| MaxRelHumidity | CONS | 1 | 93097 | -1.69 | 9.18E-02 | 0.168 |
| Vowels | MDD | 0.05 | 8160 | -1.68 | 9.23E-02 | 0.168 |
| MinSunnyDaylight | DS | 1.00E-07 | 1 | -1.67 | 9.42E-02 | 0.169 |
| LON | OPEN | 0.01 | 2016 | 1.67 | 9.49E-02 | 0.169 |
| MINPrecipRate | EXTRA | 1 | 110345 | -1.67 | 9.49E-02 | 0.169 |
| SumMaxTemp | OPEN | 1.00E-04 | 26 | -1.66 | 9.66E-02 | 0.171 |
| BacteriaDiversity | ASD | 0.01 | 3305 | -1.64 | 1.01E-01 | 0.177 |
| WinMaxTemp | OPEN | 5.00E-08 | 1 | 1.64 | 1.02E-01 | 0.177 |
| Vowels | NEUROgpc | 5.00E-08 | 1 | 1.63 | 1.02E-01 | 0.177 |
| MinSunnyDaylight | MDD | 0.1 | 15013 | -1.60 | 1.10E-01 | 0.189 |
| LON | SWB | 1.00E-04 | 79 | -1.58 | 1.13E-01 | 0.191 |
| MaxRelHumidity | SCZ | 1.00E-06 | 200 | -1.58 | 1.13E-01 | 0.191 |
| WinMaxTemp | BD | 1.00E-04 | 82 | 1.58 | 1.15E-01 | 0.192 |
| VirusDiversity | OPEN | 1.00E-06 | 2 | 1.58 | 1.15E-01 | 0.192 |
| MinSunnyDaylight | EXTRA | 1.00E-04 | 50 | 1.57 | 1.17E-01 | 0.193 |
| MINPrecipRate | NEUROssgac | 1.00E-05 | 92 | -1.56 | 1.20E-01 | 0.195 |
| MaxSunnyDaylight | NEUROssgac | 0.001 | 903 | -1.56 | 1.20E-01 | 0.195 |
| Segments | AGREE | 1 | 93096 | 1.55 | 1.21E-01 | 0.195 |
| MINPrecipRate | SCZ | 0.3 | 75011 | -1.55 | 1.22E-01 | 0.195 |
| MINPrecipRate | BD | 0.01 | 2833 | 1.55 | 1.22E-01 | 0.195 |
| Vowels | AGREE | 1 | 93096 | 1.53 | 1.26E-01 | 0.197 |
| LON | NEUROgpc | 0.01 | 3151 | -1.53 | 1.27E-01 | 0.197 |
| ProtozoaDiversity | SCZ | 1.00E-04 | 903 | 1.53 | 1.27E-01 | 0.197 |
| MinSunnyDaylight | NEUROgpc | 1 | 110359 | 1.53 | 1.27E-01 | 0.197 |
| SumMaxTemp | EXTRA | 0.001 | 436 | -1.52 | 1.29E-01 | 0.199 |
| Vowels | CONS | 1.00E-04 | 21 | -1.51 | 1.31E-01 | 0.201 |
| WinMinTemp | ASD | 5.00E-08 | 1 | 1.50 | 1.33E-01 | 0.202 |
| ALT | CONS | 1.00E-05 | 3 | -1.48 | 1.39E-01 | 0.208 |
| WinMaxTemp | ASD | 0.001 | 402 | 1.48 | 1.39E-01 | 0.208 |
| LAT | NEUROgpc | 1 | 110359 | -1.47 | 1.41E-01 | 0.210 |
| LON | DS | 0.3 | 52341 | -1.47 | 1.42E-01 | 0.210 |
| MINPrecipRate | AGREE | 1 | 93096 | 1.46 | 1.43E-01 | 0.210 |
| MaxPrecipRate | CONS | 0.001 | 221 | -1.46 | 1.44E-01 | 0.210 |
| MaxSunnyDaylight | NEUROgpc | 0.01 | 3151 | -1.46 | 1.45E-01 | 0.211 |
| LAT | MDD | 0.01 | 1938 | 1.45 | 1.48E-01 | 0.213 |
| VirusDiversity | NEUROssgac | 1 | 103553 | -1.43 | 1.52E-01 | 0.217 |
| Segments | ASD | 0.05 | 13870 | 1.43 | 1.52E-01 | 0.217 |
| MinSunnyDaylight | SWB | 0.001 | 453 | -1.43 | 1.54E-01 | 0.217 |
| MinSunnyDaylight | ASD | 1.00E-06 | 2 | 1.42 | 1.55E-01 | 0.217 |
| WinMinTemp | NEUROgpc | 5.00E-08 | 1 | 1.42 | 1.55E-01 | 0.217 |
| MaxRelHumidity | MDD | 1.00E-05 | 2 | 1.41 | 1.59E-01 | 0.221 |
| MinRelHumidity | AGREE | 1.00E-05 | 2 | -1.40 | 1.61E-01 | 0.222 |
| Consonants | DS | 1.00E-06 | 7 | 1.38 | 1.69E-01 | 0.232 |
| SumMaxTemp | SWB | 1.00E-06 | 4 | 1.37 | 1.72E-01 | 0.234 |
| MaxSunnyDaylight | CONS | 0.001 | 221 | 1.35 | 1.76E-01 | 0.239 |
| MINPrecipRate | ASD | 1.00E-06 | 2 | -1.35 | 1.78E-01 | 0.240 |
| LON | AGREE | 0.01 | 1956 | -1.33 | 1.82E-01 | 0.245 |
| ProtozoaDiversity | NEUROssgac | 0.1 | 22517 | -1.32 | 1.88E-01 | 0.249 |
| MinSunnyDaylight | NEUROssgac | 1.00E-06 | 43 | 1.32 | 1.88E-01 | 0.249 |
| Segments | NEUROssgac | 0.1 | 22517 | -1.31 | 1.89E-01 | 0.249 |
| BacteriaDiversity | NEUROgpc | 5.00E-08 | 1 | -1.31 | 1.92E-01 | 0.251 |
| MaxRelHumidity | OPEN | 1.00E-04 | 26 | 1.30 | 1.93E-01 | 0.251 |
| WinMinTemp | CONS | 1 | 93097 | -1.30 | 1.95E-01 | 0.252 |
| WinMaxTemp | CONS | 0.05 | 8354 | 1.29 | 1.97E-01 | 0.253 |
| MaxRelHumidity | BD | 0.1 | 18919 | 1.29 | 1.98E-01 | 0.253 |
| MaxPrecipRate | NEUROgpc | 0.05 | 12665 | 1.29 | 1.99E-01 | 0.253 |
| BacteriaDiversity | DS | 0.05 | 12832 | 1.28 | 2.00E-01 | 0.254 |
| ProtozoaDiversity | SWB | 0.001 | 453 | -1.26 | 2.08E-01 | 0.262 |
| Segments | SCZ | 0.01 | 8546 | 1.26 | 2.09E-01 | 0.262 |
| Segments | OPEN | 1.00E-06 | 2 | 1.24 | 2.16E-01 | 0.269 |
| SumMinTemp | NEUROgpc | 5.00E-08 | 1 | -1.23 | 2.18E-01 | 0.270 |
| MinSunnyDaylight | OPEN | 1.00E-04 | 26 | -1.23 | 2.20E-01 | 0.270 |
| SumMaxTemp | NEUROssgac | 0.001 | 903 | -1.23 | 2.20E-01 | 0.270 |
| MaxSunnyDaylight | DS | 1.00E-07 | 1 | -1.22 | 2.22E-01 | 0.270 |
| MINPrecipRate | MDD | 1.00E-05 | 2 | -1.21 | 2.26E-01 | 0.275 |
| Vowels | NEUROssgac | 1.00E-06 | 43 | -1.20 | 2.29E-01 | 0.276 |
| VirusDiversity | CONS | 0.1 | 15491 | -1.19 | 2.33E-01 | 0.278 |
| MaxSunnyDaylight | AGREE | 0.1 | 15420 | -1.19 | 2.33E-01 | 0.278 |
| ProtozoaDiversity | AGREE | 0.001 | 232 | -1.19 | 2.34E-01 | 0.278 |
| VirusDiversity | NEUROgpc | 1 | 110359 | -1.18 | 2.38E-01 | 0.282 |
| Consonants | ASD | 1.00E-04 | 41 | 1.14 | 2.56E-01 | 0.301 |
| Consonants | NEUROgpc | 1 | 110359 | 1.12 | 2.61E-01 | 0.305 |
| LAT | ASD | 0.001 | 402 | 1.11 | 2.66E-01 | 0.310 |
| SumMaxTemp | MDD | 0.1 | 15013 | -1.11 | 2.69E-01 | 0.311 |
| WinMaxTemp | NEUROgpc | 0.3 | 54772 | 1.10 | 2.70E-01 | 0.311 |
| LAT | SCZ | 0.1 | 36033 | 1.09 | 2.77E-01 | 0.316 |
| MinRelHumidity | NEUROssgac | 1.00E-07 | 18 | 1.09 | 2.77E-01 | 0.316 |
| LAT | SWB | 0.1 | 17558 | -1.08 | 2.82E-01 | 0.320 |
| ALT | ASD | 0.1 | 25330 | 1.07 | 2.84E-01 | 0.320 |
| VirusDiversity | ASD | 5.00E-08 | 1 | 1.05 | 2.93E-01 | 0.323 |
| SumMinTemp | CONS | 0.05 | 8354 | 1.05 | 2.93E-01 | 0.323 |
| MaxPrecipRate | AGREE | 0.5 | 60659 | 1.05 | 2.94E-01 | 0.323 |
| LON | BD | 0.5 | 67695 | -1.05 | 2.95E-01 | 0.323 |
| BacteriaDiversity | AGREE | 1 | 93096 | -1.05 | 2.95E-01 | 0.323 |
| LON | ASD | 0.001 | 402 | -1.05 | 2.95E-01 | 0.323 |
| MaxSunnyDaylight | OPEN | 1.00E-04 | 26 | -1.04 | 2.96E-01 | 0.323 |
| ALT | NEUROssgac | 5.00E-08 | 17 | 1.04 | 2.98E-01 | 0.324 |
| SumMinTemp | AGREE | 0.001 | 232 | 1.03 | 3.05E-01 | 0.330 |
| MinRelHumidity | NEUROgpc | 1.00E-04 | 71 | 1.01 | 3.12E-01 | 0.336 |
| MINPrecipRate | CONS | 1.00E-05 | 3 | 1.00 | 3.16E-01 | 0.338 |
| SumMinTemp | ASD | 0.1 | 25330 | -1.00 | 3.19E-01 | 0.340 |
| ProtozoaDiversity | NEUROgpc | 1.00E-04 | 71 | 0.97 | 3.33E-01 | 0.353 |
| BacteriaDiversity | CONS | 0.5 | 60620 | 0.95 | 3.40E-01 | 0.359 |
| SumMinTemp | MDD | 0.05 | 8160 | 0.91 | 3.63E-01 | 0.381 |
| SumMinTemp | NEUROssgac | 1.00E-07 | 18 | -0.84 | 4.03E-01 | 0.419 |
| Consonants | AGREE | 0.001 | 232 | -0.84 | 4.03E-01 | 0.419 |
| ProtozoaDiversity | BD | 0.01 | 2833 | -0.82 | 4.15E-01 | 0.430 |
| LON | NEUROssgac | 0.001 | 903 | -0.74 | 4.60E-01 | 0.474 |
| ALT | NEUROgpc | 0.1 | 22649 | 0.73 | 4.63E-01 | 0.475 |
| MinRelHumidity | SCZ | 1 | 145943 | -0.72 | 4.69E-01 | 0.480 |
| BacteriaDiversity | NEUROssgac | 1.00E-05 | 92 | -0.72 | 4.72E-01 | 0.480 |
| MaxPrecipRate | NEUROssgac | 1.00E-05 | 92 | -0.72 | 4.74E-01 | 0.480 |
| Segments | MDD | 0.5 | 60585 | 0.70 | 4.81E-01 | 0.484 |
| VirusDiversity | AGREE | 0.5 | 60659 | 0.70 | 4.82E-01 | 0.484 |
| LON | CONS | 0.3 | 40010 | 0.60 | 5.50E-01 | 0.550 |
