# Supplementary material for: Local adaptation in European populations affected the genetics of psychiatric disorders and behavioral traits
Source: Genome Med. 2018 Mar 26;10:24. doi: 10.1186/s13073-018-0532-7 (PMC5870256; doi:10.1186/s13073-018-0532-7)
Supplement: Supplementary file 7 — Figure S1. Null distribution generated from 10,000 random permutations of the significant PRS datasets. Blue lines represent the observed results. Abbreviations are reported in Table 1 and Table 2. (DOCX 135 kb) [file 13073_2018_532_MOESM7_ESM.docx]

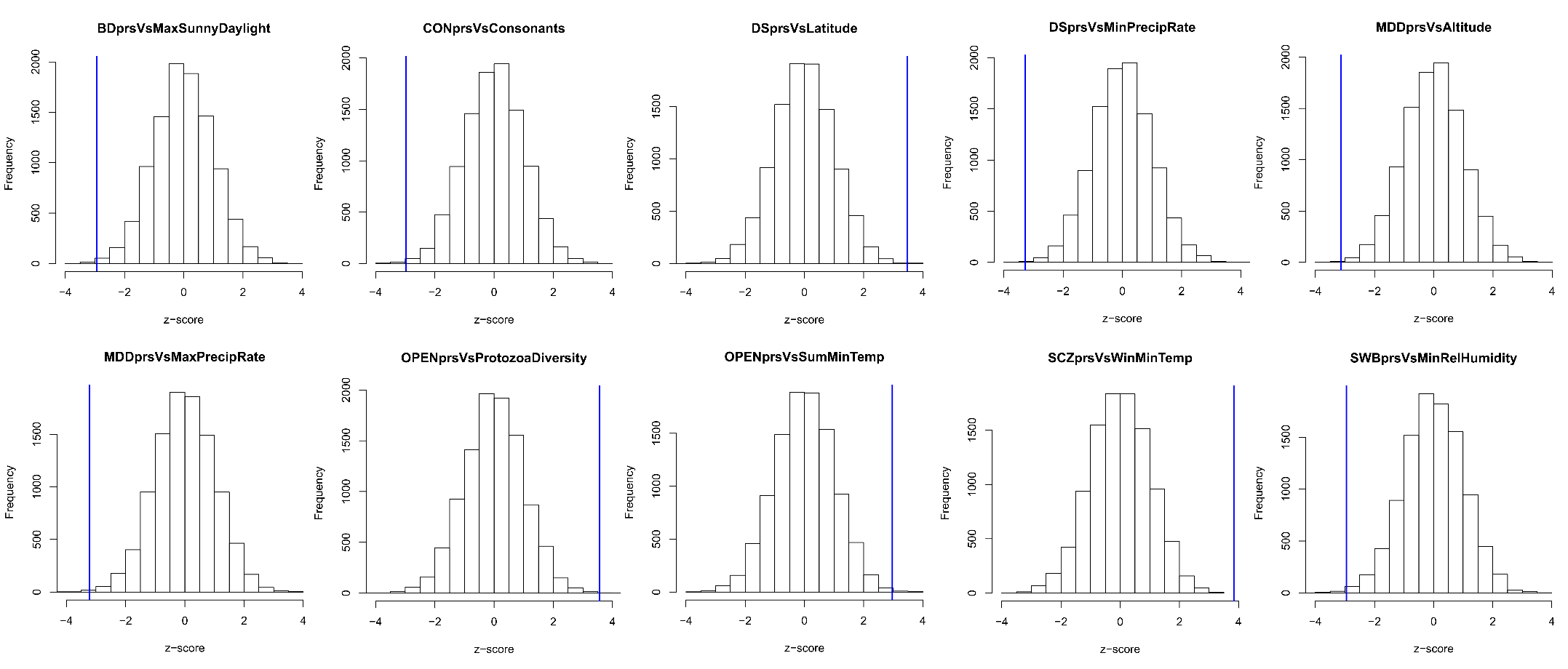


Additional file 7: Fig. S1 - Null distribution generated from 10,000 random permutations of the significant PRS datasets. Blue lines represent the observed results. Abbreviations are reported in Table 1 and Table 2.
