# Supplementary material for: Local adaptation in European populations affected the genetics of psychiatric disorders and behavioral traits
Source: Genome Med. 2018 Mar 26;10:24. doi: 10.1186/s13073-018-0532-7 (PMC5870256; doi:10.1186/s13073-018-0532-7)
Supplement: Supplementary file 8 — Table S7. Association between height PRS and local-adaptation variables. Abbreviations are reported in Table 2. (DOCX 12 kb) [file 13073_2018_532_MOESM8_ESM.docx]

**Additional file 8: Table S7** - Association between Height PRS and local-adaptation variables. Abbreviations are reported in Table 2.

| **Local-Adaptation variable** | **PT** | **SNP N** | **R2** | **P value** |
| --- | --- | --- | --- | --- |
| MIN_PrecipRate | 0.1 | 32683 | 0.21% | 0.005 |
| Min_RelHumidity | 0.3 | 58410 | 0.19% | 0.008 |
| Bacteria_diversity | 0.3 | 58410 | 0.13% | 0.020 |
| LAT | 0.05 | 22975 | 0.13% | 0.037 |
| Protozoa_diversity | 0.01 | 11191 | 0.07% | 0.038 |
| SUM_MaxTemp | 0.05 | 22975 | 0.13% | 0.047 |
| Max_RelHumidity | 0.1 | 32683 | 0.02% | 0.074 |
| Consonants | 1 | 104255 | 0.03% | 0.080 |
| Segments | 1 | 104255 | 0.05% | 0.097 |
| LON | 0.0001 | 2634 | 0.02% | 0.170 |
| Max_SunnyDaylight | 0.0000001 | 880 | 0.02% | 0.198 |
| WIN_MinTemp | 0.000001 | 1159 | 0.04% | 0.210 |
| Min_SunnyDaylight | 1 | 104255 | 0.02% | 0.267 |
| Max_PrecipRate | 0.001 | 4868 | 0.04% | 0.293 |
| SUM_MinTemp | 0.5 | 76974 | 0.02% | 0.337 |
| Altitude | 1 | 104255 | 0.02% | 0.439 |
| Vowels | 0.05 | 22975 | 0.02% | 0.439 |
| Virus_diversity | 0.0001 | 2634 | 0.01% | 0.662 |
| WIN_MaxTemp | 0.0001 | 2634 | <0.01% | 0.696 |
