# Supplementary material for: Local adaptation in European populations affected the genetics of psychiatric disorders and behavioral traits
Source: Genome Med. 2018 Mar 26;10:24. doi: 10.1186/s13073-018-0532-7 (PMC5870256; doi:10.1186/s13073-018-0532-7)
Supplement: Supplementary file 9 — Table S8. Covariate analysis of SCZ PRS with respect to winter minimum temperature (WMinTemp), winter maximum temperature (WMaxTemp), and longitude (LON). We report top PRS cutoff and cutoff obtained from the main analysis. (DOCX 12 kb) [file 13073_2018_532_MOESM9_ESM.docx]

**Additional file 9: Table S8 -** Covariate analysis of SCZ PRS with respect to winter minimum temperature (WMiT), winter maximum temperature (WMaxTemp), and longitude (LON). We report top PRS cutoff and cutoff obtained from the main analysis.

| **Local-Adaptation variable** | **PT** | **SNP N** | **R^2^** | **P value** |
| --- | --- | --- | --- | --- |
| LON | 10^-7^ | 131 | 0.01% | 0.242 |
|  | 0.5 | 104,106 | 0.01% | 0.353 |
| WMaxTemp | 0.001 | 2437 | 0.02% | **0.038** |
|  | 0.5 | 104,106 | 0.02% | 0.104 |
| WMinTemp | 10^-6^ | 200 | 0.04% | 0.061 |
|  | 0.5 | 104,106 | 0.01% | 0.252 |
