# Supplementary material for: Local adaptation in European populations affected the genetics of psychiatric disorders and behavioral traits
Source: Genome Med. 2018 Mar 26;10:24. doi: 10.1186/s13073-018-0532-7 (PMC5870256; doi:10.1186/s13073-018-0532-7)
Supplement: Supplementary file 10 — Table S9. Gene Ontology (GO) enrichment in the SCZ-WinMinTemp (Abbreviations are reported in Table 1 and Table 2) result that survived FDR multiple testing correction (q < 0.05). (DOCX 12 kb) [file 13073_2018_532_MOESM10_ESM.docx]

**Additional file 10: Table S9** - Gene Ontology (GO) enrichment in the SCZ-WMiT (Abbreviations are reported in Table 1 and Table 2) result that survived FDR multiple testing correction (q< 0.05).

| **GO ID** | **GO Term** | **SNP N** | **P value** | **Q value** |
| --- | --- | --- | --- | --- |
| GO:0008285 | negative regulation of cell proliferation | 25 | 1.01E-07 | 0.0002 |
| GO:0035567 | non-canonical Wnt signaling pathway | 5 | 3.56E-06 | 0.005 |
| GO:0017147 | Wnt-protein binding | 5 | 6.41E-06 | 0.006 |
| GO:2000041 | negative regulation of planar cell polarity pathway involved in axis elongation | 3 | 1.51E-05 | 0.010 |
| GO:0071481 | cellular response to X-ray | 3 | 2.60E-05 | 0.011 |
| GO:0090244 | Wnt signaling pathway involved in somitogenesis | 4 | 2.27E-05 | 0.011 |
| GO:0001657 | ureteric bud development | 6 | 5.39E-05 | 0.017 |
| GO:0010719 | negative regulation of epithelial to mesenchymal transition | 3 | 6.01E-05 | 0.017 |
| GO:0030308 | negative regulation of cell growth | 11 | 4.78E-05 | 0.017 |
| GO:0015693 | magnesium ion transport | 3 | 6.97E-05 | 0.018 |
| GO:0042476 | odontogenesis | 5 | 9.72E-05 | 0.021 |
| GO:0090263 | positive regulation of canonical Wnt signaling pathway | 10 | 9.65E-05 | 0.021 |
| GO:0030514 | negative regulation of BMP signaling pathway | 5 | 2.20E-04 | 0.040 |
| GO:0045668 | negative regulation of osteoblast differentiation | 5 | 2.46E-04 | 0.040 |
| GO:0045786 | negative regulation of cell cycle | 6 | 2.29E-04 | 0.040 |
| GO:0060070 | canonical Wnt signaling pathway | 7 | 2.48E-04 | 0.040 |
