# Supplementary material for: Local adaptation in European populations affected the genetics of psychiatric disorders and behavioral traits
Source: Genome Med. 2018 Mar 26;10:24. doi: 10.1186/s13073-018-0532-7 (PMC5870256; doi:10.1186/s13073-018-0532-7)
Supplement: Supplementary file 11 — Table S10. Gene Ontology (GO) enrichment in the MDD-altitude result that survived FDR multiple testing correction (q < 0.05). Abbreviations are reported in Table 1 and Table 2. (DOCX 14 kb) [file 13073_2018_532_MOESM11_ESM.docx]

**Additional file 11: Table S10 -** Gene Ontology (GO) enrichment in the MDD-Altitude result that survived FDR multiple testing correction (q< 0.05). Abbreviations are reported in Table 1 and Table 2.

| **GO ID** | **GO Term** | **SNP N** | **P value** | **Q value** |
| --- | --- | --- | --- | --- |
| GO:0007267 | cell-cell signaling | 28 | 5.74E-07 | 0.002 |
| GO:0008330 | protein tyrosine/threonine phosphatase activity | 5 | 1.72E-06 | 0.003 |
| GO:0001706 | endoderm formation | 6 | 4.04E-06 | 0.005 |
| GO:0005307 | choline:sodium symporter activity | 5 | 1.43E-05 | 0.011 |
| GO:0008292 | acetylcholine biosynthetic process | 5 | 1.43E-05 | 0.011 |
| GO:0051770 | positive regulation of nitric-oxide synthase biosynthetic process | 9 | 2.10E-05 | 0.014 |
| GO:0017147 | Wnt-protein binding | 6 | 3.61E-05 | 0.020 |
| GO:0007271 | synaptic transmission, cholinergic | 7 | 4.75E-05 | 0.023 |
| GO:0002224 | toll-like receptor signaling pathway | 18 | 8.75E-05 | 0.026 |
| GO:0007623 | circadian rhythm | 13 | 7.73E-05 | 0.026 |
| GO:0034142 | toll-like receptor 4 signaling pathway | 16 | 7.40E-05 | 0.026 |
| GO:0050729 | positive regulation of inflammatory response | 13 | 7.91E-05 | 0.026 |
| GO:0097381 | photoreceptor disc membrane | 5 | 6.48E-05 | 0.026 |
| GO:1900227 | positive regulation of NLRP3 inflammasome complex assembly | 7 | 9.18E-05 | 0.026 |
| GO:0008048 | calcium sensitive guanylate cyclase activator activity | 5 | 1.11E-04 | 0.027 |
| GO:0033265 | choline binding | 5 | 1.05E-04 | 0.027 |
| GO:0046534 | positive regulation of photoreceptor cell differentiation | 4 | 1.26E-04 | 0.029 |
| GO:0015220 | choline transmembrane transporter activity | 5 | 1.37E-04 | 0.029 |
| GO:0042562 | hormone binding | 5 | 1.51E-04 | 0.029 |
| GO:0060084 | synaptic transmission involved in micturition | 2 | 1.49E-04 | 0.029 |
| GO:0008285 | negative regulation of cell proliferation | 34 | 2.06E-04 | 0.035 |
| GO:0045671 | negative regulation of osteoclast differentiation | 8 | 2.04E-04 | 0.035 |
| GO:2000041 | negative regulation of planar cell polarity pathway involved in axis elongation | 3 | 1.94E-04 | 0.035 |
| GO:0015871 | choline transport | 5 | 2.60E-04 | 0.038 |
| GO:0034134 | toll-like receptor 2 signaling pathway | 13 | 2.52E-04 | 0.038 |
| GO:0038123 | toll-like receptor TLR1:TLR2 signaling pathway | 13 | 2.47E-04 | 0.038 |
| GO:0038124 | toll-like receptor TLR6:TLR2 signaling pathway | 13 | 2.47E-04 | 0.038 |
| GO:0003884 | D-amino-acid oxidase activity | 2 | 3.40E-04 | 0.041 |
| GO:0006275 | regulation of DNA replication | 4 | 3.37E-04 | 0.041 |
| GO:0017017 | MAP kinase tyrosine/serine/threonine phosphatase activity | 5 | 3.49E-04 | 0.041 |
| GO:0036088 | D-serine catabolic process | 2 | 3.40E-04 | 0.041 |
| GO:0055130 | D-alanine catabolic process | 2 | 3.40E-04 | 0.041 |
| GO:0071481 | cellular response to X-ray | 3 | 3.31E-04 | 0.041 |
| GO:0002755 | MyD88-dependent toll-like receptor signaling pathway | 13 | 4.26E-04 | 0.042 |
| GO:0005578 | proteinaceous extracellular matrix | 16 | 3.91E-04 | 0.042 |
| GO:0042541 | hemoglobin biosynthetic process | 3 | 4.27E-04 | 0.042 |
| GO:0046676 | negative regulation of insulin secretion | 7 | 4.28E-04 | 0.042 |
| GO:0060080 | inhibitory postsynaptic potential | 4 | 3.95E-04 | 0.042 |
| GO:0060907 | positive regulation of macrophage cytokine production | 6 | 4.04E-04 | 0.042 |
| GO:0070273 | phosphatidylinositol-4-phosphate binding | 5 | 4.31E-04 | 0.042 |
| GO:0005501 | retinoid binding | 3 | 4.54E-04 | 0.043 |
| GO:0012507 | ER to Golgi transport vesicle membrane | 3 | 4.79E-04 | 0.044 |
| GO:0050702 | interleukin-1 beta secretion | 7 | 4.96E-04 | 0.045 |
| GO:0002322 | B cell proliferation involved in immune response | 5 | 5.33E-04 | 0.047 |
| GO:0003870 | 5-aminolevulinate synthase activity | 2 | 6.40E-04 | 0.047 |
| GO:0005109 | frizzled binding | 5 | 5.48E-04 | 0.047 |
| GO:0008201 | heparin binding | 16 | 5.86E-04 | 0.047 |
| GO:0031284 | positive regulation of guanylate cyclase activity | 5 | 6.10E-04 | 0.047 |
| GO:0031295 | T cell costimulation | 3 | 6.50E-04 | 0.047 |
| GO:0031663 | lipopolysaccharide-mediated signaling pathway | 9 | 5.60E-04 | 0.047 |
| GO:0032757 | positive regulation of interleukin-8 production | 7 | 6.28E-04 | 0.047 |
| GO:0042088 | T-helper 1 type immune response | 5 | 6.10E-04 | 0.047 |
| GO:0051712 | positive regulation of killing of cells of other organism | 4 | 5.71E-04 | 0.047 |
| GO:0090244 | Wnt signaling pathway involved in somitogenesis | 4 | 6.17E-04 | 0.047 |
