# Supplementary material for: Local adaptation in European populations affected the genetics of psychiatric disorders and behavioral traits
Source: Genome Med. 2018 Mar 26;10:24. doi: 10.1186/s13073-018-0532-7 (PMC5870256; doi:10.1186/s13073-018-0532-7)
Supplement: Supplementary file 12 — Figure S2. Distribution of the results of GO enrichment analysis from 100 random sets. Orange line represents q < 0.05. (DOCX 225 kb) [file 13073_2018_532_MOESM12_ESM.docx]

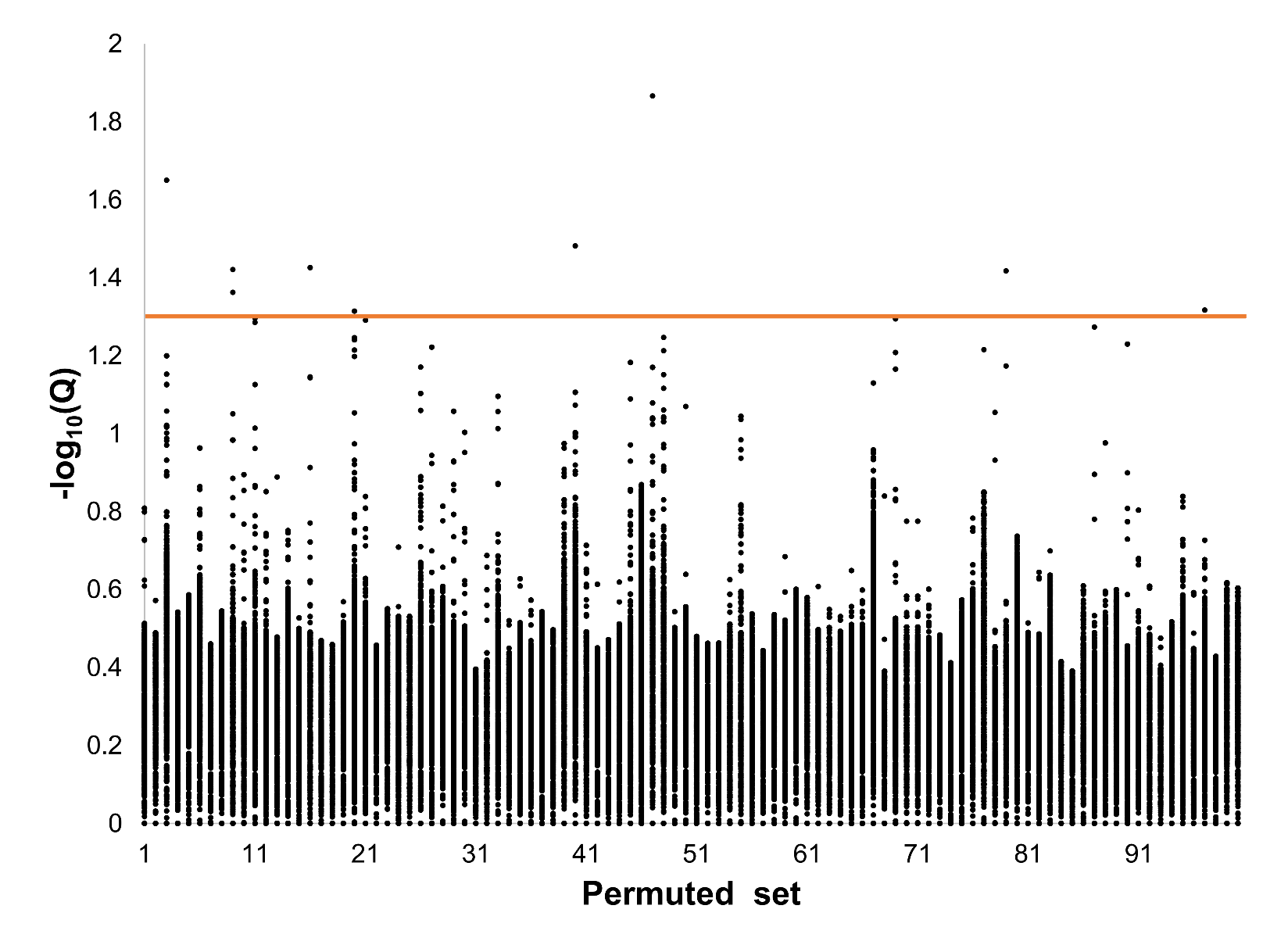


Additional file 12: Fig. S2 - Distribution of the results of GO enrichment analysis from 100 random sets. Orange line represents q < 0.05.
