# Supplementary material for: Local adaptation in European populations affected the genetics of psychiatric disorders and behavioral traits
Source: Genome Med. 2018 Mar 26;10:24. doi: 10.1186/s13073-018-0532-7 (PMC5870256; doi:10.1186/s13073-018-0532-7)
Supplement: Supplementary file 13 — Figure S3. Overall distribution of q values generated from the GO enrichment analysis of 100 random sets. The red line represents q < 0.05. (DOCX 69 kb) [file 13073_2018_532_MOESM13_ESM.docx]

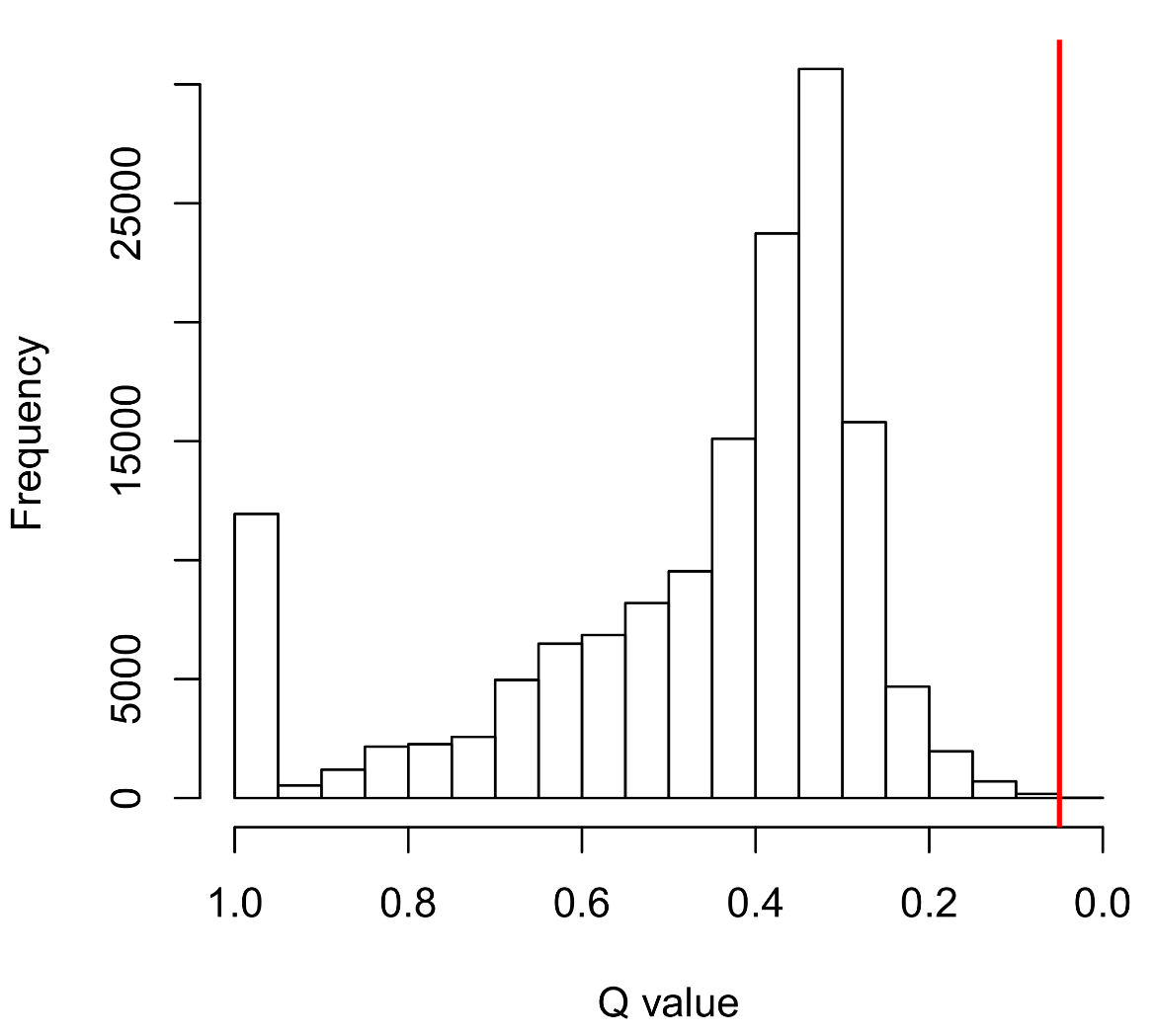


**Additional file 13: Fig. S3** - Overall distribution of Q values generated from the GO enrichment analysis of 100 random sets. The red line represents q < 0.05.
