# Supplementary figures and images for: Local adaptation in European populations affected the genetics of psychiatric disorders and behavioral traits
Source: Genome Med. 2018 Mar 26;10:24. doi: 10.1186/s13073-018-0532-7 (PMC5870256; doi:10.1186/s13073-018-0532-7)

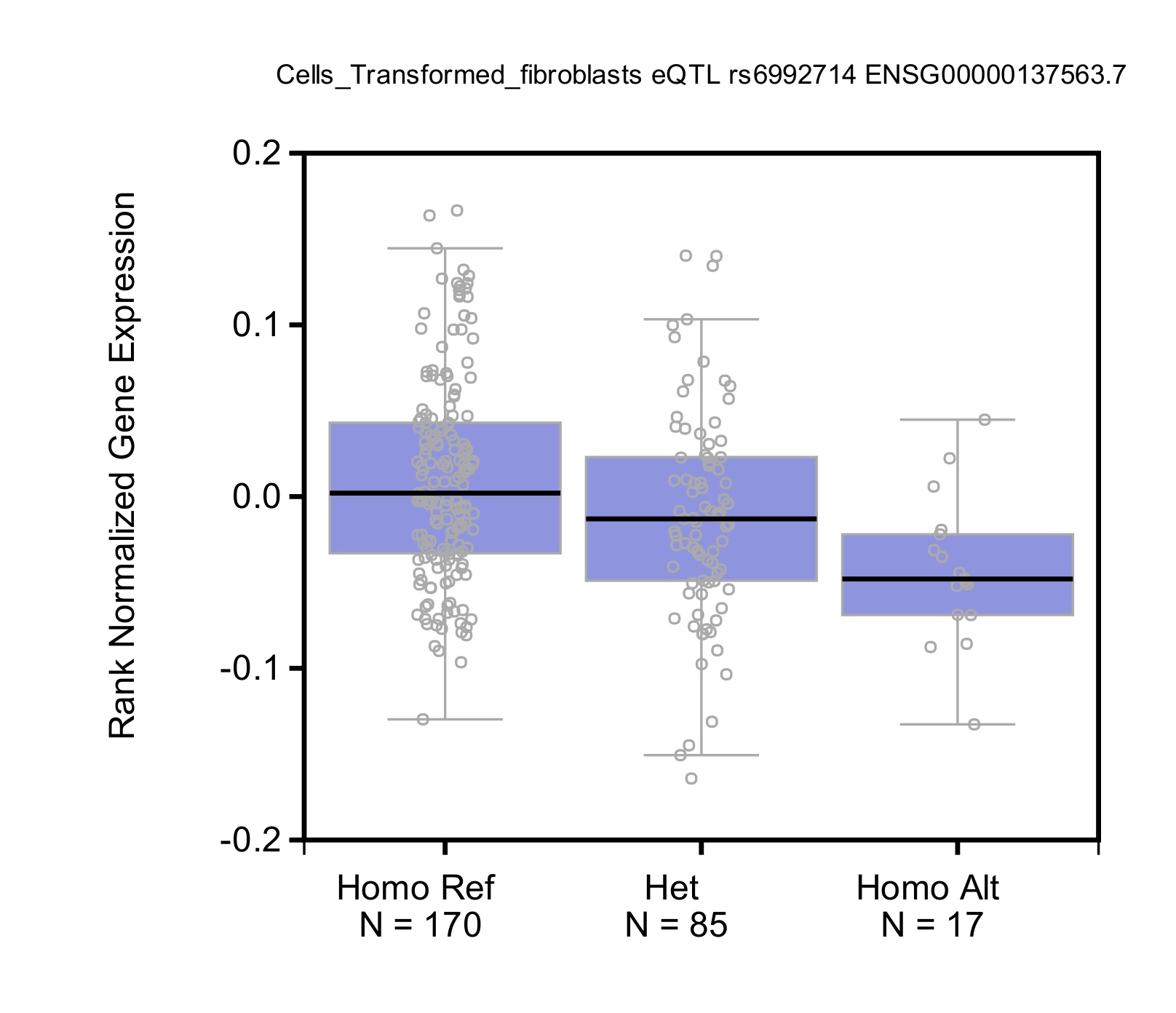


**Additional file 14: Fig. S4 -** Significant association of rs6992714 with *GGH* gene expression.

Supplement: Supplementary file 14 — Figure S4. Significant association of rs6992714 with GGH gene expression. (DOCX 140 kb) [file 13073_2018_532_MOESM14_ESM.docx]
