# Supplementary material for: Local adaptation in European populations affected the genetics of psychiatric disorders and behavioral traits
Source: Genome Med. 2018 Mar 26;10:24. doi: 10.1186/s13073-018-0532-7 (PMC5870256; doi:10.1186/s13073-018-0532-7)
Supplement: Supplementary file 15 — Table S11. Addictive effects in variants included in single-locus and oligogenic PRS from NOIA analysis. (DOCX 12 kb) [file 13073_2018_532_MOESM15_ESM.docx]

**Additional file 15: Table S11** - Addictive effects in variants included in single-locus and oligogenic PRS from NOIA analysis.

| **PRS** | **Local-Adaptation variable** | **rsID** | **Z score** | **P value** |
| --- | --- | --- | --- | --- |
| DSe-7 | Latitude | rs6992714 | 3.154 | 1.61E-03 |
|  | SumMaxTemp |  | -2.926 | 3.43E-03 |
| OPENe-6 | ProtozoaDiversity | rs10932966 | 2.357 | 1.84E-02 |
|  |  | rs1477268 | 2.802 | 5.08E-03 |
| OPENe-8 | SumMinTemp | rs1477268 | 3.028 | 2.46E-03 |
| SWBe-6 | MinRelHumidity | rs6587766 | -0.903 | 3.67E-01 |
|  |  | rs17693963 | -2.958 | 3.10E-03 |
|  |  | rs4842283 | -2.266 | 2.35E-02 |
|  |  | rs2075677 | -3.174 | 1.50E-03 |
