# Supplementary material for: Local adaptation in European populations affected the genetics of psychiatric disorders and behavioral traits
Source: Genome Med. 2018 Mar 26;10:24. doi: 10.1186/s13073-018-0532-7 (PMC5870256; doi:10.1186/s13073-018-0532-7)
Supplement: Supplementary file 16 — Table S12. Significant associations of rs10932966 with RP11-16P6.1 gene expression in multiple tissues. (DOCX 11 kb) [file 13073_2018_532_MOESM16_ESM.docx]

**Additional file 16: Table S12** - Significant associations of rs10932966 with *RP11-16P6.1* gene expression in multiple tissues.

| **Tissue** | **Effect Size** | **P value** |
| --- | --- | --- |
| Muscle - Skeletal | 0.44 | 1.20E-09 |
| Adipose - Subcutaneous | 0.34 | 1.10E-06 |
| Artery - Tibial | 0.3 | 5.10E-06 |
| Nerve - Tibial | 0.3 | 1.30E-05 |
| Skin - Sun Exposed (Lower leg) | 0.25 | 1.60E-05 |
| Lung | 0.23 | 2.20E-05 |
| Thyroid | 0.18 | 4.00E-05 |
